# Natural history of the narrow endemics *Ipomoea cavalcantei* and *I*. *marabaensis* from Amazon Canga savannahs

**DOI:** 10.1038/s41598-017-07398-z

**Published:** 2017-08-08

**Authors:** Elena Babiychuk, Sergei Kushnir, Santelmo Vasconcelos, Mariana Costa Dias, Nelson Carvalho-Filho, Gisele Lopes Nunes, Jorge Filipe dos Santos, Lourival Tyski, Delmo Fonseca da Silva, Alexandre Castilho, Vera Lucia Imperatriz Fonseca, Guilherme Oliveira

**Affiliations:** 1 Instituto Tecnológico Vale, Rua Boaventura da Silva 955, Bairro Nazaré, CEP 66055-090 Belém, Pará Brazil; 2 Parque Zoobotânico Vale, VALE S.A., Rod. Raimundo Mascarenhas, Km 26, S/N., Núcleo Urbano de Carajás, CEP 68516 000 Parauapebas, Pará Brazil; 3 VALE S.A., Rua Guamá N°60, Prédio DIFN, Núcleo Urbano de Carajás, CEP: 68516-000 Parauapebas, Pará Brazil

## Abstract

Amazon comprises a vast variety of ecosystems, including savannah-like Canga barrens that evolved on iron-lateritic rock plateaus of the Carajás Mountain range. Individual Cangas are enclosed by the rain forest, indicating insular isolation that enables speciation and plant community differentiation. To establish a framework for the research on natural history and conservation management of endemic Canga species, seven chloroplast DNA loci and an ITS2 nuclear DNA locus were used to study natural molecular variation of the red flowered *Ipomoea cavalcantei* and the lilac flowered *I*. *marabaensis*. Partitioning of the nuclear and chloroplast gene alleles strongly suggested that the species share the most recent common ancestor, pointing a new independent event of the red flower origin in the genus. Chloroplast gene allele analysis showed strong genetic differentiation between Canga populations, implying a limited role of seed dispersal in exchange of individuals between Cangas. Closed haplotype network topology indicated a requirement for the paternal inheritance in generation of cytoplasmic genetic variation. Tenfold higher nucleotide diversity in the nuclear ITS2 sequences distinguished *I*. *cavalcantei* from *I*. *marabaensis*, implying a different pace of evolutionary changes. Thus, Canga ecosystems offer powerful venues for the study of speciation, multitrait adaptation and the origins of genetic variation.

## Introduction

Plants present remarkable opportunities for studying adaptive radiation and speciation in action^1^. Interactions with pollinators and herbivores^2^, adaptation to soils and harsh environmental conditions^3^ are among the major ecological factors that drive adaptive radiation in plants. Ecogeographic factors such as mountain range uplifts and island formation set gene flow restrictive reproductive isolation barriers in plants and facilitate rapid speciation rates^4^. Speciation by hybridization is another very common process in plants^4, 5^.

**Figure 1:**
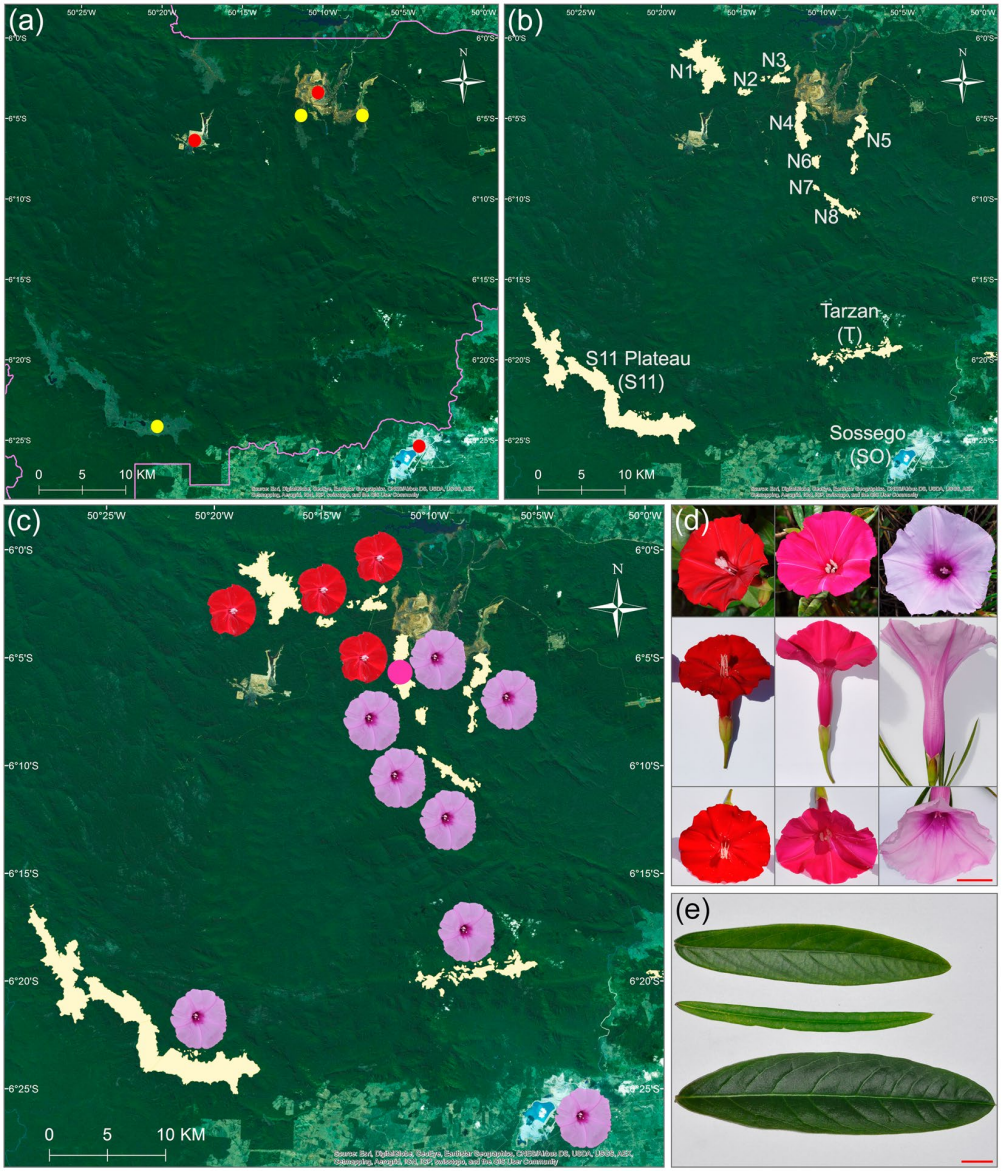
(**a**) Map of the Carajás National Forest. Forest boundary is indicated with the purple line. The patchy light green areas are deforestation areas used for agriculture and cattle pastures. Intense green color is a mountain rain forest. Paler green areas within the Forest are savannahs and granitic inselbergs. Established open-pit mines and the ongoing mine explorations are indicated by the red and yellow dots, respectively. (**b**) Location of the populations used in this study. **(c)** Distribution of *I*. *cavalcantei* (red flowers) and *I*. *marabaensis* (lilac flowers). The purple dot indicates the location where putative interspecies hybrids were found in 2016. **(d)** From left to right, flower of a representative *I*. *cavalcantei* individual, flower of a putative hybrid, and flower of *I*. *marabaensis*. Bar = 2 cm. **(e)** Leaves from *I*. *cavalcantei* (upper leaf, from Canga N4), *I*. *marabaensis* (lower leaf, from S11 Plateau). Individuals of *I*. *marabaensis* from populations N5, N6, N8 and Tarzan have narrow lanceolate leaves (middle leaf). Bar = 1 cm.

The Carajás Mountain range is located in the south-eastern lowland Amazon. Part of the mountain range is protected within the Carajás National Forest (Fig. 1a,b). Rock weathering in Carajás Mountains and suitable climatic conditions, such as distinct wet and dry seasons, led to the localized formation of iron-laterite rocks at the surface of small plateaus elevated 650–800 m above sea level^6^. Plateaus host fire prone savannah-like ecosystems known as Canga that are isolated from each other by the rain forest^7^ (Supplementary Fig. S1). Canga soils are shallow (0–10 cm), unevenly distributed though rock fissures and landscape depressions; are edaphically restrictive and often contain potentially phytotoxic levels of metals^8, 9^. The rain fall precipitation varies through the dry-wet seasons between <60 mm to 1900 mm/month. Average monthly temperatures vary between 19 and 31 °C. Fires are common. Thus, low nutrients, heat, openness, drought prone and toxic metal rich conditions in combination with the insular isolation resulted in highly specialized Canga plant communities composed of more than 500 plant species^9, 10^.

The iconic plant of Carajás, known as “flor de Carajás” - “flower of Carajás” in Portuguese, is a red flowered morning glory *Ipomoea cavalcantei* ^11^ from the family Convolvulaceae that comprises commonly known food crop sweet potatoes, which is *Ipomoea batatas*. *I*. *cavalcantei* is a narrow endemic that to the best of our knowledge is only found in Carajás National Forest at four northern Canga islands, measuring at most 20 km^2^ (Figs 1c and S2; Table S1). The lilac flowered *I*. *marabaensis* ^12^ is a related species that inhabits, but is not restricted to Cangas south and east from *I*. *cavalcantei* (Figs 1c and S2; Table S1). The leaf morphology distinguishes populations of *I*. *marabaensis* ^12^ (Fig. 1e). The flower traits differentiation between *I*. *cavalcantei* and *I*. *marabaensis* suggests adaptive radiation driven by pollinator preferences^2^. *I*. *cavalcantei* bright red tubular flowers with protruding reproductive organs are reminiscent of a hummingbird pollination syndrome, whereas large flowers of *I*. *marabaensis* with a broad tube display the features of a bee pollination syndrome^13^ (Fig. 1d). The assumed shift in pollinator preferences should favor the species co-occurrence in the same area, i.e. sympatry. Why *I*. *cavalcantei* and *I*. *marabaensis*, adapted to Canga environments, have very distinct areal is not understood. The herbarium of the Museum Emilio Goeldi (Belém, Pará, Brazil) holds specimens that were classified as interspecies *I*. *cavalcantei* × *marabaensis* hybrids^14^. There are no reports of the cytogenetic or molecular characterization of the putative natural hybrids (Fig. 1d), as well as their role in a gene flow and/or incipient speciation.

In the present study, seven chloroplast DNA loci and an ITS2 nuclear DNA locus were used to study natural molecular variation of *I*. *cavalcantei* and *I*. *marabaensis* in Cangas of the Carajás National Forest. Soil transplantation experiments with *I*. *cavalcantei*, *I*. *marabaensis* and four other Convolvulaceae species were carried out. The primary goals were to: (1) investigate the genetic structure and diversity of *I*. *cavalcantei* and *I*. *marabaensis* populations; (2) find molecular evidence for the hypotheses that *I*. *cavalcantei* and *I*. *marabaensis* are recently diverged, sister species^12^ that could hybridize in nature; (3) study the role of Canga soils in structuring plant communities and species distributions; (4) establish a framework for the future research on natural history and conservation management of Carajás morning glories.

## Results

### *I*. *cavalcantei* and *I*. *marabaensis* belong to a lineage within the clade Murucoides

To decipher the phylogenetic affinities of *I*. *cavalcantei* and *I*. *marabaensis* using molecular markers, partial coding sequences of seven genes encoded by the plastomes (cpDNA) were determined and compared to respective sequences computationally extracted from the assembled and sequenced plastomes of twenty six *Ipomoea* species^15^. Maximum parsimony, maximum likelihood and Bayesian likelihood analyses of the concatenated sequence alignments positioned *I*. *cavalcantei* and *I*. *marabaensis* within the clade of Murucoides (Fig. S3). In addition, alignments of *psbA-trnH* intergenic spacers showed that species from the Murucoides clade have a large deletion, i.e. approximately 150–190 bp as compared to the species from clades Pes-caprae and Quamoclit (Fig. S4).

### *I*. *cavalcantei* and *I*. *marabaensis* share chloroplast DNA polymorphisms

cpDNA sequence analysis revealed molecular polymorphisms in *rpoC1* and *psbA-trnH* intergenic (IGS) sequences (Table S2). The C/A single nucleotide polymorphism (SNP) in a coding region of *rpoC1* was nonsynonymous, alternating the AAA codon for the positively charged lysine (K) and CAA codon for the hydrophilic noncharged glutamine (Q) amino acid residues. It is unlikely that plastidial RNA editing converts CAA codon to a translation termination codon UAA, thus we infer that rpoC1^K^ and rpoC1^Q^ subunit isoforms characterize the plastidial prokaryotic type RNA polymerase in *I*. *cavalcantei* and *I*. *marabaensis*. In the plant kingdom, rpoC1 polypeptides from many lineages are characterized by the K/Q variation at this site, e.g. among the top 35 hits in a blast-p search 19 species had K and 16 had Q. However, within the family Convolvulaceae, species such as *Convolvulus arvensis*, *Turbina corymbosa*, *Operculina macrocarpa*, *Merremia quinquefolia*, *Evolvulus nuttallianus*, *Argyreia nervosa*, *Cuscuta reflexa*, as well as twenty six *Ipomoea* species^15^ all had rpoC1^K^ isoform, suggesting that rpoC1^K^ is an ancestral state in morning glories, whereas the emergence of rpoC1^Q^ in *I*. *cavalcantei* and *I*. *marabaensis* could be an example of convergent evolution in a 687 amino acid residues long rpoC1 polypeptide. Analysis of available 26 Ipomoea *rpoC1* sequences showed additional SNP variation at 17 sites, of which two were nonsynonymous resulting in hydrophobic amino acid residue variation L/V and V/I.

The *psbA-trnH* IGS from *I*. *cavalcantei* and *I*. *marabaensis* individuals were identical in length, but differed in a sequence at three neighboring positions, i.e. AAA versus TTT.

**Figure 2:**
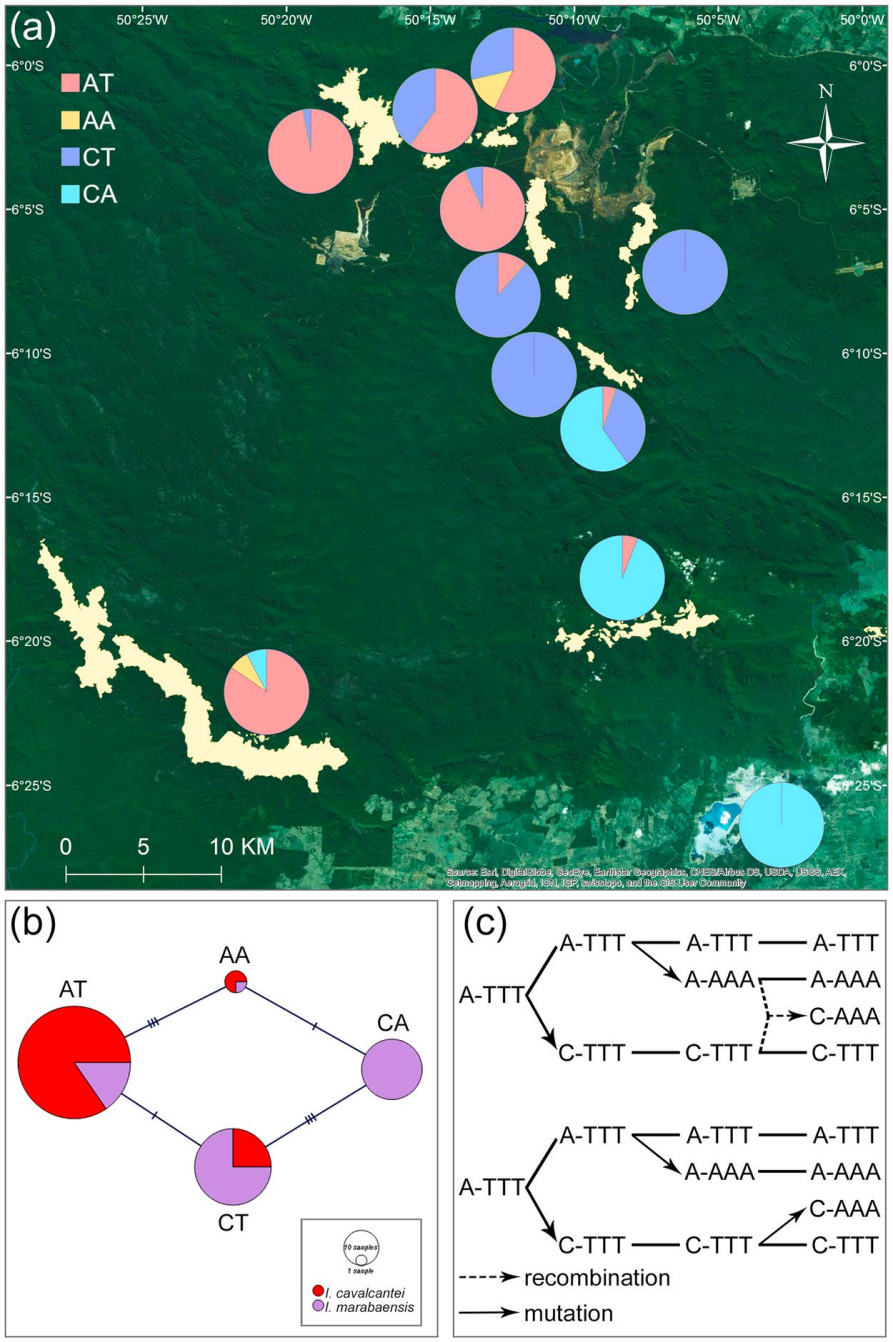
**(a)** Distribution of the four plastome types of Carajás morning glories. **(b)** Parsimony network of plastome haplotypes. Bars across the network edges indicate the numbers of mutational changes. **(c)** The mutation and recombination scenarios of plastome diversity origin among ancestors of the species. Plastome types/haplotypes are represented by polymorphic sites identified in *rpoC1* (A/C SNP) and *psbA-trnH* (AAA/TTT SNP stretch) sequences. Dash is a several thousand base pairs long gap between the genes. Plastome AT (A-TTT) was arbitrary chosen as ancestral.

We assumed that the gene order in *Ipomoea* spp. plastomes is conserved, accordingly the polymorphisms at *rpoC1* and *psbA-trnH* IGS were phased into haplotypes that identify at least four plastome types in *I*. *cavalcantei* and *I*. *marabaensis* (Fig. 2a; Table S3). The plastome type network was closed (Fig. 2b), i.e. all possible assortment of polymorphisms into four plastome haplotypes have been found in wild populations of *I*. *cavalcantei* and *I*. *marabaensis*. Observed haplotypes could have originated among ancestors of the species either from three mutational changes, i.e. mutation origin scenario, or two mutations and one recombination event, i.e. recombination origin scenario (Fig. 2c).

The CA plastome was only identified among *I*. *marabaensis* individuals in this study, whereas three other plastome types occurred in both species. We cannot exclude that the observed absence of CA plastome in *I*. *cavalcantei* could be due to its very low frequencies in the species and was not captured by the population sampling in this study. The *rpoC1* ^*Q*^ and *rpoC1* ^*K*^ were predominant alleles in *I*. *marabaensis* (66 *rpoC1* ^*Q*^/17 *rpoC1* ^*K*^) and *I*. *cavalcantei* (12 *rpoC1* ^*Q*^/91 *rpoC1* ^*K*^), respectively. The overall interspecies haplotype diversity *h* and nucleotide diversity π calculated over 883 bp of *rpoC1* and *psbA-trnH* sequences were higher in *I*. *marabaensis* (*h* = 0.6560; π = 0.001997), than in *I*. *cavalcantei* (*h* = 0.2581; π = 0.000429) (see also the results of the basic population statistic calculations for individual Cangas in Table S4).

Calculation of pairwise *F*_*ST*_ values indicated significant genetic differentiation among populations (Table 1). Surprisingly, the *I*. *marabaensis* samples collected at S11 Plateau Canga were more similar to *I*. *cavalcantei* populations (*F*_*ST*_ range 0.011–0.115) than to the conspecific *I*. *marabaensis* populations from the Northern Cangas N6, N7 and N8 or South-Eastern Canga Tarzan (*F*_*ST*_ range 0.632–0.807) (Table 1), most probably due to the abundance of the plastome type AT (Fig. 2a).

**Table 1:**
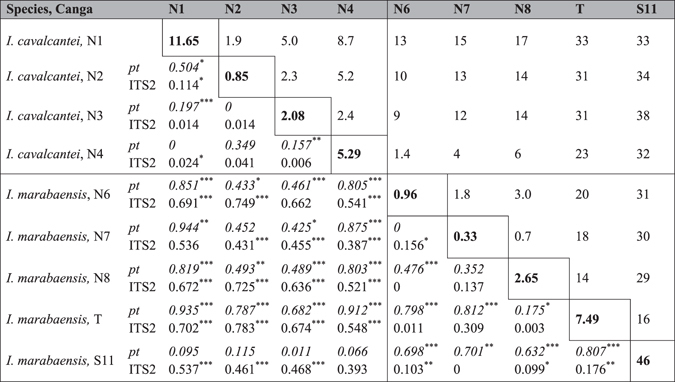
Canga areas, geographic distances and genetic differentiation of *I*. *cavalcantei* and *I*. *marabaensis* populations. The values along diagonal (in bold) are areas of Cangas (km^2^). Values above diagonal are the shortest distances between Canga islands (km). The pairwise genetic differentiation measured by the population descriptive statistics is shown below the diagonal. The *F*_*ST*_ values from the plastome (*pt*) gene alleles, emphasized by italics font, and internal transcribed rDNA spacer alleles (ITS2) are combined in the same cells. Asterisk are the *p* values: ^*^<0.05; ^**^<0.01; ^***^<0.001.

AMOVA analysis within *I*. *cavalcantei* suggested that 13.92% of variation can be explained by the genetic differentiations between Canga populations (Table 2). AMOVA analysis of eleven Canga group permutations indicated that most of the variation between populations could be due to the differences between the Canga N1 & N4 group *versus* Canga N2 & N3 group (Table 2). This population differentiation scenario was also supported by the analysis using software package STRUCTURE^16^ (Fig. S5). The differentiation of Canga N3 (n = 21) could be due to the three individuals with AA plastome, that was not found in N1 (n = 33) or N4 (n = 44) (Table S3). The AMOVA results do not appear to correlate with geographic distances, because only 1.9 km separate N1 from N2 and 2.4 km N3 from N4, whereas the closest distance between N1 and N4 is approximately 8.7 km (Table 1).

**Table 2:** AMOVA results, partitioning molecular variation of plastomes.

Species	Variance component	*df*	Variance %	Fixation index	*P*
*I*. *cavalcantei* & *I*. *marabaensis* ^a^	Among species	1	44.74	Φ_CT_ = 0.447	<0.05
	Among populations within species	7	28.71	Φ_SC_ = 0.519	<0.001
	Within populations	174	26.55	Φ_ST_ = 0.734	<0.001
*I*. *cavalcantei* ^b^	Among populations	3	13.92	Φ_ST_ = 0.139	<0.01
	Within populations	99	86.08		
*I*. *cavalcantei* ^c^	Among population groups	1	24.05	Φ_CT_ = 0.241	0.341
	Among populations within groups	2	0	Φ_SC_ = 0	0.654
	Within populations	99	75.95	Φ_ST_ = 0.229	<0.01
*I*. *marabaensis* ^d^	Among populations	4	64.01	Φ_ST_ = 0.64	<0.001
	Within populations	75	35.99		
*I*. *marabaensis* ^e^	Among population groups	2	62.47	Φ_CT_ = 0.625	0.086
	Among populations within groups	2	5.65	Φ_SC_ = 0.151	<0.05
	Within populations	75	31.87	Φ_ST_ = 0.681	<0.001

^a^Canga groups (N1, N2, N3, N4), (N6, N7, N8, T, S11).

^b^One group of Canga populations (N1, N2, N3, N4).

^c^Canga populations groups (N1, N4), (N2, N3).

^d^One group of Canga populations (N6, N7, N8, T, S11).

^e^Canga population groups (N6, N7), (N8, T), (S11).

AMOVA (Table 2) and STRUCTURE (Fig. S5) analyses of *I*. *marabaensis* populations suggested the plausible structure N6 & N7 *vs* N8 & Tarzan *vs* S11 Plateau. The northern populations N6, N7 and perhaps N5 (the latter was only represented by 2 individuals and not included in AMOVA analyses) have predominant plastome CT, the south-eastern group of Cangas N8, Tarzan and a single individual on a granitic inselberg Sossego mainly have plastome type CA. The plastome type AT was predominant (11 plants out of 13 analyzed) in S11 Plateau Canga (Table S3).

### Diversification of transcribed internal ribosomal RNA gene spacer

**Figure 3:**
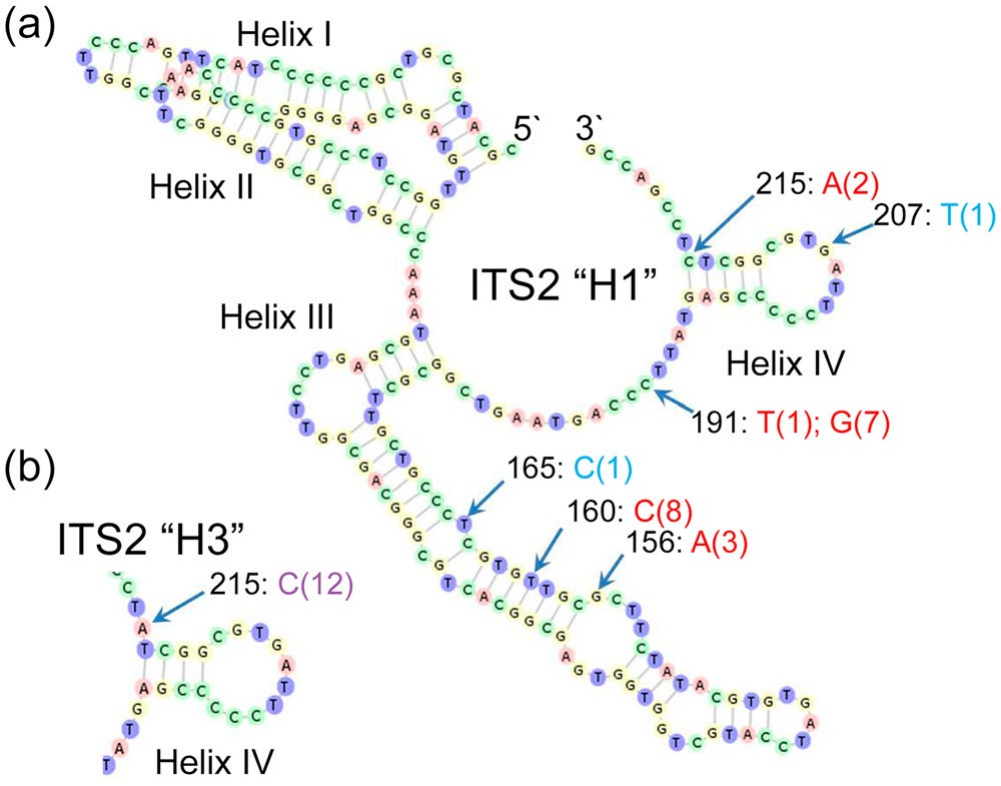
Distribution of SNP’s over the ITS2 secondary structure. **(a)** ITS2 haplotype “H1” and **(b)** the Helix IV of the folded ITS2 haplotype “H3”. Arrows point bases at which natural variation was observed. Numbers in black are the bases numbering from the 5′end. The letters next to arrows are the alternative bases, i.e. SNP; bracketed numbers are instances among fourteen recorded haplotypes. SNP are colored in red, when a polymorphism is only found in *I*. *cavalcantei*; in blue for *I*. *marabaensis*; in purple, when shared between the species.

Next, we characterized the ribosomal RNA gene (rDNA) internal spacer ITS2 that is a standard type of the nuclear genome loci used in plant phylogenetic analysis and population genetics studies^17, 18^. In all 217 analyzed sequences we did not detect insertions-deletions, thus ITS2 regions had the same length of 223 base pairs. Six positions that are clustered within 67 bp window closer to the 28S rRNA coding region showed SNP variation (Fig. 3). There was a species-specificity in distribution of polymorphic sites. Four sites varied in *I*. *cavalcantei* and two in *I*. *marabaensis* (Fig. 3a; Tables S5 and S6). Base 215 represented a polymorphic site that was shared between the species. This polymorphism was the only one that affected the length of the base-pairing in a helix IV (Fig. 3b). Analysis of more than fifty *Ipomoea* ITS2 sequence-structures at ITS2 Database revealed numerous insertion/deletion (indel) polymorphisms, as well as SNP’s distributed all over the *Ipomoea* ITS2. The ITS2 sequences of *I*. *polpha*, *I*. *arborescens*, *I*. *carnea* and *I*. *conzattii*, that could belong to Murucoides clade, were more similar to *I*. *cavalcantei* and *I*. *marabaensis* ITS2 and showed only two 1 base pair indels. Eighteen polymorphic SNP sites differentiated those species from *I*. *cavalcantei* and *I*. *marabaensis* in the upstream ITS2 region that comprise Helixes I and II, suggesting certain constrains on *I*. *cavalcantei/I*. *marabaensis* ITS2 polymorphisms distribution, which could result from co-evolvement of the rRNA precursor sequences and the cognate RNA processing machinery^19^.

**Figure 4:**
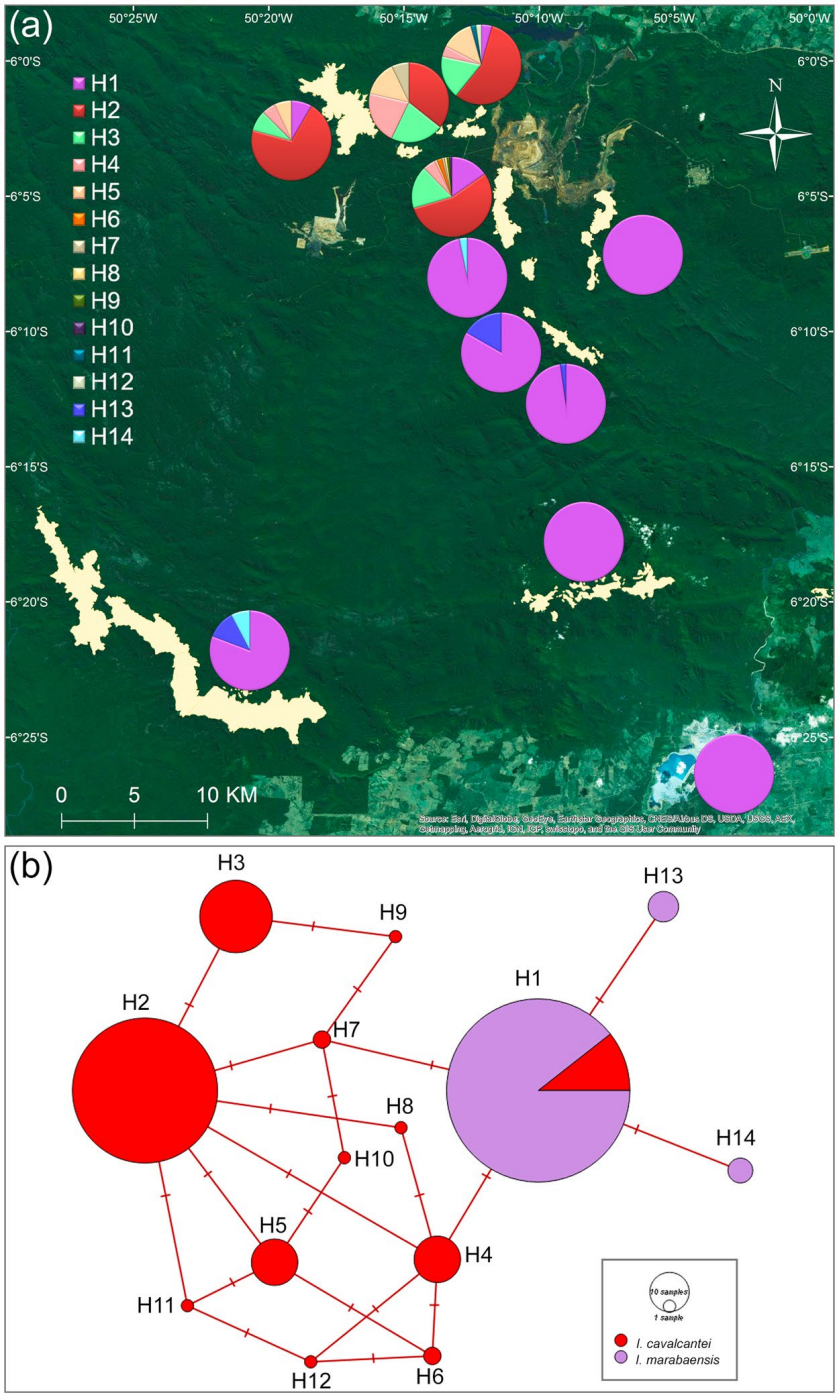
Nuclear genome ITS2 **(a)** allele distribution and **(b)** haplotypes network.

We identified fourteen ITS2 haplotypes, of which eleven were found only in *I*. *cavalcantei*, two only in *I*. *marabaensis*. One haplotype H1 was shared between the species (Fig. 4a; Tables S5 and S6). Quantification of this variation showed six to ten-fold interspecific differences in haplotype and nucleotide diversities, *I*. *cavalcantei* (*h* = 0.6172; π = 0.004092); *I*. *marabaensis* (*h* = 0.1186; π = 0.000543), a situation opposite to the plastome diversity estimates (see the results of the basic population statistic calculations for individual Cangas in Supplementary Table S7). The ITS2 haplotype H1 was predominant in *I*. *marabaensis*. It could be either ancestral, or indicate a gene flow between the species^20, 21^.

Maximum parsimony network modelling showed that all identified ITS2 haplotypes could derive from each other by single base pair substitutions (Fig. 4b). Interestingly, several *I*. *cavalcantei* haplotypes formed closed sub-networks, indicating gene recombination/conversion followed by rDNA repeat homogenization as a mechanism of their origin within heterozygous individuals^20, 22^. Indeed, the average observed heterozygosity in *I*. *cavalcantei* was five times higher than in *I*. *marabaensis* (Table S7).

The *F*_*ST*_ pairwise differences between *I*. *cavalcantei* populations showed little genetic differentiation (Table 1). Moderate genetic differentiation characterized some of *I*. *marabaensis* populations. In this species, S11 Plateau Canga was well differentiated from N6, N8 and T, which fits its separation by the distance. However, a small (0.33 km^2^) 30 km distant Canga N7 was not differentiated from S11 (*F*_*ST*_ = 0), but differentiated from N6 (*F*_*ST*_ = 0.156) and N8 (*F*_*ST*_ = 0.137) that are separated from N7 by 1.8 and 0.7 km, respectively.

AMOVA indicated that most of genetic variation in *I*. *cavalcantei* and *I*. *marabaensis* originates from ITS2 genetic differences within the populations (Table 3). AMOVA population analyses also showed the higher inbreeding levels in *I*. *marabaensis* (*F*_*IS*_ = 0.78–1), as compared to *I*. *cavalcantei* (*F*_*IS*_ = 0.31–0.59). ITS2 molecular data clustering using STRUCTURE revealed a clear interspecies differentiation, but, consistently with AMOVA analysis, indicated the absence of intraspecific genetic differentiation (Fig. S6).

**Table 3:** AMOVA results, partitioning molecular variation in ITS2.

Species	Variance component	Variance %	Fixation index	*P*
*I*. *cavalcantei* & *I*. *marabaensis* ^a^	Among species	58.86	Φ_CT_ = 0.588	<0.01
	Among populations within species	1.05	Φ_SC_ = 0.026	<0.01
	Within populations	40.07	Φ_ST_ = 0.599	<0.001
*I*. *cavalcantei* ^b^	Among populations	2.39	Φ_ST_ = 0.0233	<0.05
	Within populations	97.67		
*I*. *cavalcantei* ^c^	Among population groups	0.22	Φ_CT_ = 0.002	0.319
	Among populations within groups	2.2	Φ_SC_ = 0.022	0.061
	Within populations	97.6	Φ_ST_ = 0.024	<0.05
*I*. *marabaensis* ^d^	Among populations	7.67	Φ_ST_ = 0.0767	<0.001
	Within populations	92.33		
*I*. *marabaensis* ^e^	Among population groups	3.91	Φ_CT_ = 0.039	0.365
	Among populations within groups	4.5	Φ_SC_ = 0.045	<0.05
	Within populations	91.58	Φ_ST_ = 0.084	<0.05

^a^Canga groups (N1, N2, N3, N4), (N6, N7, N8, T, S11).

^b^One group of Canga populations (N1, N2, N3, N4).

^c^Canga populations groups (N1, N4), (N2, N3).

^d^One group of Canga populations (N6, N7, N8, T, S11).

^e^Canga population groups (N6, N7), (N8, T), (S11).

### Molecular analysis of the putative hybrids

Flow cytometry analysis showed that *I*. *cavalcantei* and *I*. *marabaensis* have similar genome sizes (Table S8), presenting no evidence that differentiation in ploidy or genome size could establish barriers to gene flow between the species. The putative *I*. *cavalcantei* × *marabaensis* hybrids had a genome size like parental species, indicating that those phenotypically distinct individuals (Fig. 1d) are not autopolyploids or allopolyploids.

A single available *I*. *marabaensis* plant from a sympatric area in Canga N4 had plastome type CT and ITS2 haplotype H1, which is consistent with the allele frequencies in the closest Canga N6 *I*. *marabaensis* population. Three putative interspecies hybrid plants from the same area all had plastome type AT, suggesting *I*. *cavalcantei* as a mother, if we assume maternally-biased cytoplasmic inheritance in the species, although a reversion from maternal inheritance mode to paternal has been observed in interspecies crosses of *Passiflora* ^23^. All putative hybrids had ITS2 haplotype H1, which is shared between *I*. *cavalcantei* and *I*. *marabaensis*. Thus, available data did not conclusively demonstrate a hybrid nature of analyzed individuals.

### Differential plant growth on Canga soils

**Figure 5:**
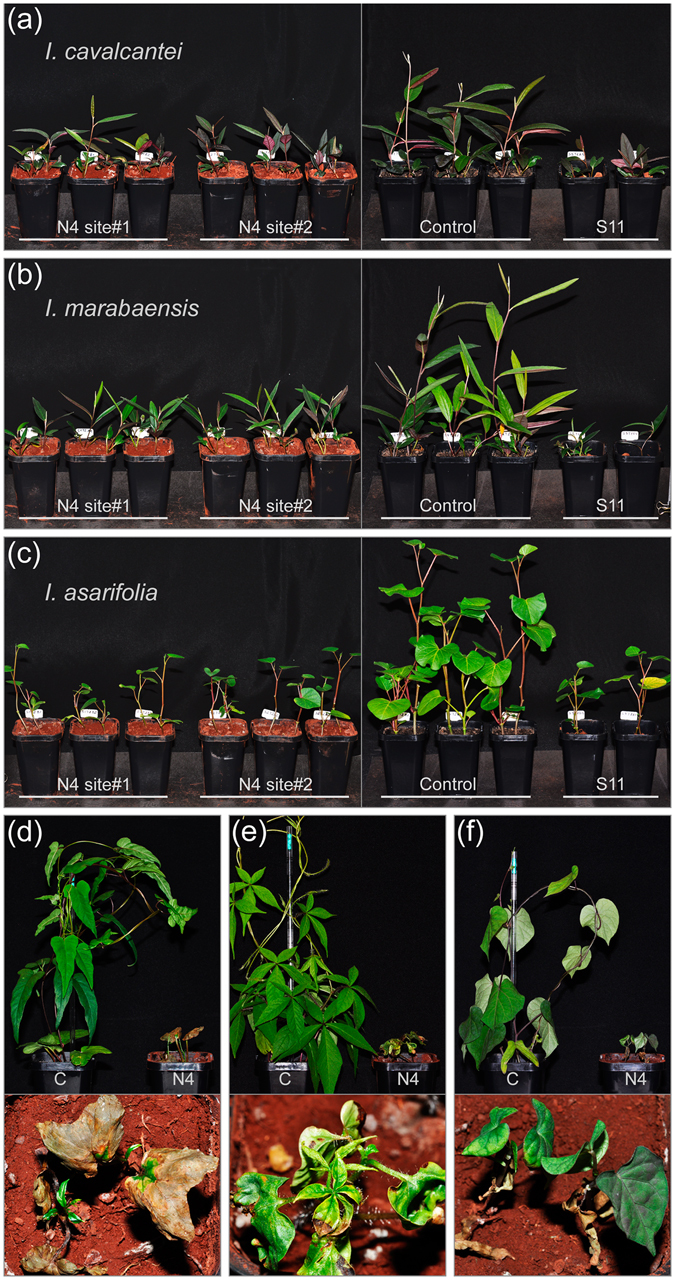
The growth of **(a)** *I*. *cavalcantei*, **(b)** *I*. *marabaensis*, and **(c)** *I*. *asarifolia* on soils collected from Canga N4 (N4 site#1, N4 site#2), S11 Plateau Canga (S11), and horticultural soil (Control). Pictures taken ten weeks after germination. *Operculina hamiltonii* **(d)**, *Merremia aegyptia* **(e)** and *Ipomoea grandifolia* **(f)** grown on horticultural soil (C) or Canga soil (N4). Seedlings grown on Canga soil (N4) were retarded, shed leaves that developed necrotic lesions (lower close up images).

*I*. *cavalcantei* and *I*. *marabaensis* ten-week-old plantlets produced 0.27 g (±0.059 s.d.) and 0.24 g (±0.06 s.d.) of the dry leaf biomass, respectively (Fig. S7), when grown on horticultural soil that had high levels of available nutrients (Table S9). The leaf biomass produced on three nutrient-rationed Canga soils, which had lower contents of major nutrients, such as P, S, K, Ca^+2^, Mg^+2^ (Table S9), was 20–30% (*I*. *cavalcantei*) and 17–22% (*I*. *marabaensis*) of the biomass produced on horticultural soil (Figs 5a,b and S7), indicating an absence of specific species dependence on Canga soils and potential suitability of abandoned agricultural land for *ex situ* conservation.

*Operculina hamiltonii* is common in edaphically restrictive, very dry Carajás granitic inselberg ecosystem, which is similar to Canga (Fig. S1). However, seedlings of *O*. *hamiltonii* grown on Canga soils showed little shoot growth, whereas both cotyledon leaves and young shoots developed extensive necrotic lesions. After the death of the primary shoot, the development of the new side shoots was as well aborted soon after emergence (Fig. 5d). Similar degree of growth inhibition, shoot tissue necrosis and leaf abscission characterized seedlings of *Merremia aegyptia* (Fig. 5e) and *Ipomoea grandifolia* (Fig. 5f). Unexpectedly, *Ipomoea asarifolia* seedlings grown on Canga soils produced leaf biomass that was 19–25% of leaf biomass produced by the species on horticultural soil (Fig. 5c and S7). The tested here *Ipomoea asarifolia* accession originated from a population found on rocky barrens in Atlantic coastal areas very remote from Cangas (>600 km). The species occurrence within Carajás National Forest ecosystems has not been reported, indicating species preadaptation to Canga soils.

## Discussion

We show that *I*. *cavalcantei* and *I*. *marabaensis* are closely related species from a lineage within the morning glory clade Murucoides. Studies of the *Ipomoea* L. showed that the Murucoides clade includes species with vastly different morphologies and biogeographic distributions^15, 24, 25^. Australian endemic *I*. *polpha* is a ground trailing vine, that develops tuberous roots harvested by the indigenous people for food. *I*. *murucoides* and *I*. *pauciflora* are deciduous trees that grow in arid regions of Central America. The neotropical *I*. *carnea* and *I*. *cuneifolia* are erect shrubs. Thus, it appears that the genetic makeup of the Murucoides ancestors enabled high adaptability of the derived modern species to very different environments, in particular, arid and edaphically restrictive semi-deserts, savannahs and rocky mountainous ridges.

Sequence analysis in this study suggested a Central American endemic *I*. *populina* as one of the closest relatives to *I*. *cavalcantei* and *I*. *marabaensis*. Similar to *I*. *cavalcantei*, *I*. *populina* has obovate leaves and grows as a deciduous climbing woody stemmed liana. Austin noted similarities between *I*. *cavalcantei*, *I*. *argentea* and a red flowered *I*. *longistaminea* O’Donnel from the Brazilian Cerrado and Caatinga ecosystems^11^. In addition to *I*. *longistaminea*, there are several other woody Caatinga *Ipomoea* species that develop edible tuberous roots for which they are known as mountain potatoes, “batata da serra” in Portuguese^26^. Clearly, the higher phylogenetic resolution of *I*. *cavalcantei* and *I*. *marabaensis* lineage is required to reconstruct the speciation history and to detect possible gene-flow mediated sharing of adaptive gene alleles, which could have facilitated colonization of Cangas by the morning glories.

Our results show overlaps in gene allele distributions between *I*. *cavalcantei* and *I*. *marabaensis*, which indicates that the species are sisters^12^, sharing the most recent common ancestor. Thus, *I*. *cavalcantei* could represent a new example of independent origin of red flowers in morning glories. It is generally accepted that the blue/purple flower color due to cyanidin accumulation is ancestral in *Ipomoea* L.^27^, implying that flowers of *I*. *marabaensis* are closer to the ancestral type. The reverse transitions from red to blue flowers are unlikely^27, 28^. Parsimony reconstructions indicated that red-flowered lineages have arisen four times independently in the *Ipomoea* tribe Astripomoeinae, i.e. in the lineages leading to *I*. *quamoclit/I*. *hederifolia*, *I*. *udeana*, *I*. *conzattii* and *I*. *horsfalliae* ^27^. The *I*. *conzattii* and *I*. *horsfalliae* are likely to belong to Murucoides clade and have evolved the shared red flower phenotype through different genetic mechanisms^27^. Therefore, it is possible that red flowers in the Murucoides clade independently originated at least three times. Plants employ different strategies to color flowers in red^27, 29^. Further biochemical and genetic studies of *I*. *cavalcantei* will advance our understanding of mechanisms that underpin convergent evolution of the red flower trait in plants.

Analysis of plastomes revealed significant genetic differentiation of *I*. *cavalcantei* and *I*. *marabaensis* populations, indicating the combined effects of genetic drift and population bottlenecks as mechanisms of population evolvement in Cangas. In contrast, little spatial structure was displayed by the distribution of the nuclear ITS2 polymorphisms. Thus, natural variation of plastome and ITS2 sequences suggested alternative demographic histories of *Ipomoea* populations in Carajás Cangas, which could reflect differences in gene allele dispersal by the seed or/and pollen^18, 30^. Such contrasting genetic differentiations indicate maternally-biased plastome inheritance that limits plastome gene flow to the seed dispersal^30^. Although empiric studies suggest that the mode of plastome inheritance cannot always be extrapolated within a genus^31^, strict maternal inheritance in Japanese morning glory *Ipomoea nil* was inferred from the genetic analysis of a cytoplasmic mutation^32^. However, *I*. *nil* pollen sperm cells have plastids^33^, indicating a potential for biparental inheritance like in ca. 20% of angiosperm species^33, 34^. Furthermore, the rates of paternal plastome transmission, the so-called paternal leakage, increased among the offspring of interpopulation crosses in *Helianthus verticillatus* ^31^ and *Campanulastrum americanum* ^35^. The reversal from maternal to paternal plastome inheritance was found in interspecies hybrids of *Passiflora* ^23^. Thus, the potential plasticity of the plastome inheritance modes in *I*. *cavalcantei* and *I*. *marabaensis* species cannot be disregarded and should be studied further both in laboratory and natural settings.

We show that plastome haplotype network of *I*. *cavalcantei* and *I*. *marabaensis* has closed topology. We consider two scenarios to explain this property (Fig. 2c). The mutation scenario requires a repetitive origin for either *rpoC1* or *psbA-trnH* polymorphism in the species ancestry, which implies a strong selective pressure at a given polymorphic site. Only about twenty polymorphisms in rbcL amino acid residue sequences are responsible for the most cases of positive selection in CO_2_-fixing Ribulose-1,5-bisphospate carboxylase/oxygenase (Rubisco)^36^. Positive selection at Rubisco correlates with adaptation to altitude variation^37^ or aquatic environments^38^. The *rpoC1* or *psbA-trnH* polymorphisms could affect plastid gene expression. The recombination scenario is consistent with a neutral evolution mode, but requires: (i) heteroplasmy due to biparental inheritance, or paternal leakage; and (ii) encounter of the different parentage chloroplast DNA molecules within the same cellular compartment by the plastid fusion, which is thought to be a rare event in a plant life cycle^34^. Common axiomatic assumptions in plant plastome evolutionary studies are uniparental inheritance, powerful functional constraints, and absent sexual recombination^39^. However, plastome recombination was detected in artificial interspecies somatic cell hybrids^34^ and proposed as the source of the markedly different phylogenies; unusual patterns of plastome polymorphism and linkage disequilibrium in *Silene* ^40^, *Picea* ^41^, *Pinus* ^42^ and *Cycas* ^43^. Thus, closed topologies of the plastid haplotype networks could be a signature of plastome sexual recombination in a history of plant taxa.

The closed subnetworks in a nuclear genome ITS2 locus that characterize *I*. *cavalcantei* can be explained by recombination/gene conversions between the parental ITS2 copies. The unexpected finding was a greater diversity in species-specific ITS2 alleles of *I*. *cavalcantei* (eleven species-specific alleles), as compared to *I*. *marabaensis* (two species-specific alleles). It could be that the species differ in the death/birth rates of repetitive rDNA operons that comprise ITS2 sequences^44^. Importantly, speciation events are likely to be accompanied by the bursts of repeat amplifications, which is reflected in the abundance of the species-specific repeat families that discriminate closely related plant species. The toxicity of metal ions often includes genotoxicity^45^. Thus, metal rich soils could influence the spontaneous rates of mutations and recombination^46^. We provide an evidence for the toxicity of Canga soils. Comparative analysis of spontaneous mutation rates in *I*. *marabaensis* versus *I*. *cavalcantei*, and assessment of Canga soil effects on genome stability will advance our understanding of repetitive sequence dynamics in Carajás morning glories.

The Canga soils tested in the laboratory conditions were found to be restrictive for the seedling establishment of Convolvulaceae family species *Merremia aegyptia*, *Ipomoea grandifolia* and *Operculina hamiltonii*, the latter two species occur in the Carajás National Forest. The result is consistent with a proposal that soil properties are the main drivers of vegetation composition and structure in Canga^47^. The symptoms of tissue necrosis in *O*. *hamiltonii*, *M*. *aegyptia* and *I*. *grandifolia* seedlings resembled calcium deficiency, which could be exacerbated by the aluminum toxicity^48^. In line with the foliar damage symptoms, composition analysis of the tested Canga soil samples showed lower Ca^+2^ contents as compared to horticultural soil (0.1–1.4 cmol/dm^3^ *vs* 6.9 cmol/dm^3^). However, soil fertility depends on complex and often poorly understood interactions between the biological, chemical and physical properties of soil^3, 49^. Further experiments are required to decipher causality between Canga soil properties and arrest *O*. *hamiltonii*, *M*. *aegyptia* and *I*. *grandifolia* seedling growth. It also remains to be seen whether soil differentiation in Canga islands drives largely allopatric distribution of *I*. *cavalcantei* and *I*. *marabaensis*.

We show that *I*. *asarifolia* pregerminated seeds developed on Canga soils into the plantlets that produced leaf biomass quantities that were comparable to those of *I*. *cavalcantei* and *I*. *marabaensis* plantlets, indicating that Canga ecosystems of the Carajás National Forest potentially could be occupied by the invasive species. In the Serra do Rola Moça State Park (Minas Gerais State, Brazil), 60% of grassy fields in Canga biomes has been invaded by the African grass *Melinis minutiflora*, which is thought to constitute a severe threat to the biodiversity^50^. Many species of *Ipomoea* are noxious weeds^51^ of which *I*. *asarifolia* is an emerging problem in Amazon and Unites States. We searched for and identified *Ipomoea asarifolia* road-side populations at ca. 5 km distances from the Carajás National Forest boundaries. Thus, the Canga conservation management must include continuous monitoring for and eradication of the invasive species in mining areas and along interconnected road system that infringed historical Canga island isolation by the forest.

Molecular analyses of population sampling in this work showed that some polymorphisms could be restricted to specific locations. For example, *I*. *cavalcantei* ITS2 haplotypes H6, H9, H8 and H10 were only observed among Canga N4 individuals; whereas plastome type AA was only found in Canga N3 individuals. Large areas of vegetation cover of Canga N5, Canga N4 and the southern part of the Canga S11 Plateau were^12^ and are lost due to mining that sustains the global economic growth. *I*. *cavalcantei* is thought to be endemic to Carajás Canga ecosystems^10^. It is difficult to predict whether the loss of hidden molecular diversity in Canga N4 will be detrimental to the species long-term survival in Cangas N1, N2 and N3 that are currently protected. Here, we show that species can be maintained in *ex situ* collection on standard horticultural soils. One outcome of our work is an *ex situ* collection of several hundred living *I*. *cavalcantei* and *I*. *marabaensis* individuals in our laboratories. Noteworthy, we also established clonal propagation of unique genotypes. Thus, reintroduction^18^ of the lost in a wild unique genotypes and/or rare gene alleles into the less disturbed Canga islands can become an element of the species conservation management.

## Methods

### Study organisms

Species of the several genera of the family Convolvulaceae populate Carajás National Forest^14^, including the genus of morning glories *Ipomoea* L. that is the largest within family Convolvulaceae with estimated 650 species^15^. *Ipomoea cavalcantei* ^11^ and *Ipomoea marabaensis* ^12^ were the major focus of this work. The surveyed populations and species sampling are listed in a Table S1. Taxonomical treatment of the American morning glories positioned *I*. *cavalcantei* and *I*. *marabaensis* within an ill-defined, unnamed series containing sixty one species from the section Eriospermum, subgenus Eriospermum^52^. Species are similar in vegetative morphological traits (Fig. S2), indicating that they could share the ancestry. However, *I*. *cavalcantei* and *I*. *marabaensis* are likely to be differentiated by the pollination syndromes (Figs 1d and S2). Such species pairs could provide useful models for studies of speciation and adaptive gene pleiotropy^13, 51^. *I*. *cavalcantei* and *I*. *marabaensis* are adapted to similar habitats that characterize Canga savannahs (Fig. S1), however *I*. *marabaensis* range is broader and those species were also found on granitic inselbergs (Fig. S1), for example. In Carajás National Forest, species ranges are altered by the mining industry (Fig. 1a). The molecular diversity information necessary for conservation planning and management is not available.

It is thought that *I*. *cavalcantei* and *I*. *marabaensis* could hybridize in a sympatry area in Canga N4^14^. In 2016, we found four *I*. *marabaensis* individuals among the abundance of *I*. *cavalcantei* in Canga N4. No *I*. *cavalcantei* were found in Canga N6 that is only 1.4 km away from N4. In the sympatric area of Canga N4, our survey identified three putative hybrid individuals. Open anthers of putative hybrid plants had abundant pollen like *I*. *cavalcantei*, indicating proper meiosis and pollen development. We could collect a few viable seeds from three hybrids. It remains to be seen whether the progeny is from self-fertilization or cross-pollination by *I*. *cavalcantei*. However, fifty four controlled reciprocal pollinations between *I*. *cavalcantei* and *I*. *marabaensis* individuals that grew in a wild failed, indicating certain genetic or environmental constrains to interspecies hybridization.

The anthropogenic perturbation of Canga savannahs provides opportunities for invasive species, among which many Convolvulaceae species are known noxious weeds^51^. The effects of Canga soils on survival of three potentially invasive Convolvulaceae species: *Ipomoea asarifolia*, *Ipomoea grandifolia* and *Merremia aegyptia* (Table S10), has been tested. Because *I*. *marabaensis* can survive in the extreme environments of both Canga savannahs and granitic inselbergs, it was relevant to understand the invasive potential of Convolvulaceae species the distribution of which at present is limited to Carajas granitic inselbergs. Thus, the *Operculina hamiltonii* (Fig. S1; Table S10) was studied as well.

### Geographic maps of the locations

The geographic maps were generated with ArcGIS version 10.2 (www.esri.com) based on satellite imagery source (http://goto.arcgisonline.com/maps/World_Imagery): Esri, DigitalGlobe, GeoEye, Earthstar Geographics, CNES/Airbus DS, USDA, USGS, AEX, Getmapping, Aerogrid, IGN, IGP, swisstopo, and the GIS User Community. The map images were processed in Adobe Photoshop CS6 to indicate agricultural and mining activities (Fig. 1a); species ranges (Fig. 1b and c); and gene allele distributions (Figs 2 and 4).

### Plant material, DNA extraction, PCR amplification and DNA sequencing

DNA was extracted from the leaf tissues that were collected and stored in a NaCl-saturated 2% CTAB solution^53^. The DNA extractions were performed following the automated QIAcube HT (QIAGEN) protocol, using the QIAamp 96 DNA kit (QIAGEN), with some modifications. For each sample, a piece of approximately 30 mg of leaf tissue was cut, placed in a racked 1.2 mL polypropylene tube, frozen overnight at −80 °C (~18 h) and then macerated with two 3 mm carbide tungsten beads in the TissueLyser II (QIAGEN) for 1 min at 30 Hz. The powdered tissue was lysed in 600 µL of 2% CTAB lysis buffer [2% CTAB, 0.1 mM Tris-HCl (pH 8.0), 20 mM EDTA (pH 8.0), 1.4 M NaCl]. The tubes were gently mixed and incubated for 40 min at 60 °C in a water bath, followed by a 1 min spin at 4000 rpm, and then 300 µL of the supernatant were collected and transferred to a 96 square deep well plate, in which the automated extraction was carried out.

The PCR conditions for each of the analyzed regions (partial coding sequences of the *rpoB*, *rpoC1*, *rbcL* and *matK* and the intergenic spacers *atpF*-*atpH*, *psbK*-*psbI* and *psbA*-*trnH*) were set according to Shaw *et al*.^54^, with some modifications: 1.5 µL of the extracted DNA, 1.25 µL of the 10 × reaction buffer [100 mM Tris-HCl (pH 8.3) and 500 mM KCl], 1.2 µL of 25 mM MgCl_2_, 1.0 µL of the dNTP mix (2 mM each), 0.25 µL of each the forward and reverse primers (10 pmol) and 0.5 U of *Taq* polymerase, in a 12 µL reaction volume. The ITS2 amplification reactions were additionally supplemented with 1 µL of DMSO. Cycling conditions were the same for all amplicons, consisting of an initial denaturation at 94 °C for 3 min, followed by 30 cycles of 1 min denaturation at 94 °C, 1 min annealing at 54 °C (except for the *matK* reactions, in which the annealing temperature was 46 °C) and 1 min extension at 72 °C, and a final extension at 72 °C for 7 min. After the purification of the PCR products, 2 µL of the amplified DNAs were used in the bi-directional cycle sequencing reactions with the BigDye Terminator v3.1 kit (Applied Biosystems) using the manufacturer’s instructions. Amplicons were sequenced in a ABI 3730 Genetic Analyzer (Applied Biosystems). Primer sequences can be found in Supplementary Table S11.

### Sequence datasets, alignments, secondary structure, phylogenetic and genetic variation analyses

It has been proposed that *I*. *cavalcantei* and *I*. *marabaensis* could be sister species^12^. It is expected that sister species could share gene alleles due to the incomplete sorting of alleles present in the ancestor^21, 55^. Hence, we initiated characterization of the natural molecular variation in *I*. *cavalcantei* and *I*. *marabaensis*. For that purpose, plastome (cpDNA) sequences of seven genes from several individuals were analyzed^56^. The plastome sequences at *rpoB*, *rbcL*, *matK*, *psbK-psbI* and *atpF-atpH* loci were identical (Table S2). Polymorphisms characterized *rpoC1* and intergenic *psbA-trnH* sequences, hence the frequencies of those polymorphisms were analyzed in a larger number of individuals (Table S2). The genetic differentiation of *I*. *cavalcantei* and *I*. *marabaensis* was also characterized by sequence analysis of the nuclear ribosomal RNA gene (rDNA) internal spacer ITS2^17, 18, 20^. The ITS2 analyses and discussions assumed that all analyzed ITS2 sequences are derived from the homogenized rDNA operon repeats positioned at a single locus that also functions as a nucleolar organizer. It is well possible that this assumption is an oversimplification of the actual rDNA genes organization in *I*. *cavalcantei* and *I*. *marabaensis* nuclear genomes^57^. To support taxa identification, the ITS2 and cpDNA sequences *Ipomoea asarifolia*, *Ipomoea grandifolia*, *Merremia aegyptia* and *Operculina hamiltonii* were also analyzed (Table S10).

DNA sequences were edited and trimmed using the software package Geneious version 10^58^. Five datasets were generated. In one dataset, only partial protein-coding sequences of the plastome genes *rpoC*, *psbK*, *rbcL*, *matK* and *rpoB* were concatenated. This dataset did not require significant additional editing of gaps with an exception of an in-frame 30 base pairs deletion in *I*. *murucoides matK*. After removal of matK indel variation, all sequences had equal length 2767 bp. In the second set the, *atpF-trnH* sequences were added, i.e. 3086 bp long for *I*. *cavalcantei/marabaensis*. Other concatenates were different in length due to very high indel variation of the *atpF-trnH* IGS (Fig. S4). Finally, the full set had added sequences of *psbA-trnH IGS* sequences (3532 bp for *I*. *cavalcantei/marabaensis*). The derived subsets were prepared, in which gaps due to indels were deleted manually. Four plastome types that characterize *I*. *cavalcantei* and *I*. *marabaensis* were included in the analyses. The gene orthologs from *Ipomoea* spp. and an outgroup species *Merremia quinquefolia* were extracted from published complete plastome sequences^15^. Sequences were aligned using the software MUSCLE^59^. The maximum likelihood bootstrap analyses sampling 1000 pseudoreplicates was performed using the software RAxML version 8.2.7^60^ and the software PHYML^61^. Bayesian analyses were performed using MrBayes version 3.2.6^62, 63^. A maximum parsimony bootstrap analysis was performed in the program PAUP^*^ v40b10^64^.

*Ipomoea* nuclear rDNA amplicon sequences were annotated^65^ and ITS2 regions were extracted using the software available at ITS2 Database website. ITS2 were analyzed for direct folding^66^. Sequences with a direct fold were saved as possible templates. For the ITS2 sequences that did not fold directly, the best modeling template was searched amongst a pool of structures comprising *Ipomoea* ITS2 sequences reported in ITS2 Database and *I*. *cavalcantei* and *I*. *marabaensis* ITS2 that folded directly. The ITS2 haplotype H1 that is shared between *I*. *cavalcantei* and *I*. *marabaensis* was found to be the best template. Structured sequence alignments were analyzed in the alignment and editing tool 4SALE^67, 68^, which was also used to generate the images of the ITS2 secondary structures. Haplotype assignments were done manually. The ITS2 sequences that had no ambiguities indicated homozygous plants. ITS2 sequences from some individuals showed ambiguities. To infer the SNP phasing into haplotypes, we verified whether ambiguous SNP positions could be explained by hybridization between homozygous parents (Tables S5 and S6).

A minimum spanning network tree depicting the relationships of the haplotypes was produced using TCS computer program^69, 70^. Calculations of the standard diversity metrics, *F*-statistics and Analysis of Molecular Variance (AMOVA) were carried out using an integrated software package for population genetics data analysis Arlequin^71^. Genetic differentiation *F*_*ST*_ values and AMOVA were done for a single internal transcribed spacer rDNA (ITS2) locus differentiated by 14 alleles and for two plastome loci represented by two alleles each. The nucleotide diversity (π), Tajima’s *D* and Fu’s *F*_*S*_ were calculated using DNA sequences of nuclear genome locus *ITS2* (223 bp); plastome loci *rpoC1* (501 bp) and *psbA-trnH* (382 bp). In AMOVA analysis, the variation was first estimated in a single group that comprised four populations of *I*. *cavalcantei*, or five populations of *I*. *marabaensis*. Next, eleven group permutations were analyzed. The intraspecific population group assemblies that gave variation distribution similar to that of a single species group were considered to be biologically significant. We also assessed the genetic relationships by clustering of molecular data with the software package STRUCTURE version 2.3.4^16^. The datasets were analyzed in four models with or without admixture; with or without consideration of the sampling locations as prior information to assist the clustering^16^. Twenty replicate runs were conducted for every value of K between 1 and the number of locations plus 3, with a burn-in of 10.000 Markov Chain Monte Carlo (MCMC) steps followed by 10.000 or 50 000 iterations. To determine the optimal number of clusters (K), ΔK values were calculated^72^.

### Genomic size analysis

To determine whether *I*. *cavalcantei*, *I*. *marabaensis* and putative interspecies hybrids have similar genome sizes, and thus are likely to have similar ploidy, we used flow cytometry^51^. The nuclei suspensions were prepared essentially as described^73^ with a modification in the composition of the lysis buffer, which included the non-ionic detergent Triton X-100 and the phenolics absorbent poly-vinyl-pyrrolidone (PVP-40), 1% final concentrations each. The nuclei were stained with propidium iodide (PI) at a final concentration 50 µg/ml. Nuclei were obtained from fresh leaf tissues of plants grown in the laboratory. The procedure steps of leaf maceration, lysate clearing by filtration through 30 µm nylon mesh, and storage of nuclei suspensions were done on ice. For the comparative analyses, equal amounts (100 mg) of leaf tissues from two individuals were processed in 1 ml of extraction buffer. Nuclei suspensions were analyzed on a BD FACS Aria II flow cytometer, using a 488 nm laser. For each specimen, 1000 nuclei were acquired and three runs were made per sample. PI fluorescence mean was collected under 585/42 bandpass filter. The nuclei of *Petroselinum crispum* served as a reference standard (2 C = 4.50 pg)^74^.

### Plant growth and soil test experiments

To understand the role of soils both in a natural species distribution and in susceptibility of Canga ecosystems to invasive plants, we analyzed Convolvulaceae species seedling establishment on top soils. Twenty to twenty five kilograms of a mixture of the organic matter and degraded lateritic rocks to the depth of ca. 0–20 cm, referred to as “soil”, were collected at sites near growing *I*. *cavalcantei* (two sites at Canga N4) or *I*. *marabaensis* (one site at S11 Plateau Canga). The control soil commonly used for horticultural applications was purchased from a local supplier Yamanaka Comércio Ltda. (Belém, Pará, Brazil). This soil is successfully used as a gardening potting soil for growing common ornamentals, such as hibiscus, bougainvillea, several species of palms etc. This quality of the control soil was satisfactory for our purpose to determine whether *I*. *cavalcantei* and *I*. *marabaensis* could be easily maintained in *ex situ* collections.

The major goal of the experimental design was to reduce the effect of all the environmental variables, except the composition of the plant growth substrate. To eliminate the effect of the soil biotic components, soil samples were autoclaved. Equal amounts of soil by weight were distributed into plastic pots, measuring 6 × 6 × 9,5 cm. One pot was placed on a balance and water was added until the first drop of water leaked from the bottom of the pot. The same amount of water minus 5 g was then added to all pots of a given soil sample. Thus, the water content was close to the water holding potential for each soil type. The water losses due to evaporation and plant evapotranspiration were replenished every day by bringing the weight of each individual pot to its original value. In this watering regime, there was no leaching of elements from the growth substrates, because water was never leaking out of the pots. Bi-distilled deionized water (resistance 16 MOhms) was used in experiments. To facilitate synchronous germination, seeds were scarified with a sanding paper and pre-germinated on wet paper disks in Petri plates. Seeds with just emerged roots were planted on soil. Two seeds per pot were planted in triplicate. Plants were cultured at 25 °C/22 °C with 12/12 hours light/dark photoperiod in a walk-in plant growth room. The selection and identification of the control Convolvulaceae species is explained in a Supplementary Table S9. Accessions of *I*. *cavalcantei* from Cangas N1, N3, N4; and *I*. *marabaensis* from Cangas N8, Tarzan, Plateau S11 were tested in these experiments. To assess plant growth, leaves was collected, dried at 65 °C and weighted (Fig. S7). The dried leaf sample comprised leaves from two plants grown on a soil in a single pot. Nine samples per species were measured for soils from N4 site#1, N4 site #2 and Control (i.e. 9 pots with a given soil sample, 18 individuals per species). Due to limited amount of soil from S11 Plateau Canga, six dried leaf samples per species were measured.

The extractable nutrient composition and properties of the soil samples were analyzed at the Center of Agricultural and Environmental Technologies (Centro de Tecnologia Agrícola e Ambiental (CAMPO), http://www.campo.com.br/cta/), following standardized procedures developed by the Brazilian Agricultural Research Corporation (Empresa Brasileira de Pesquisa Agropecuária, EMBRAPA)^75^. Organic matter (O.M.) and organic carbon content measurements were done according to Leite *et al*.^76^; and extractable phosphorus content on anion exchange resin according to Camargo *et al*.^77^. The measured parameters and respective values are summarized in a Table S9.

## Electronic supplementary material

BABIYCHUK_SUPPLEMENTAL INFORMATION_SREP-16-51438B
