# Supplementary material for: Natural history of the narrow endemics Ipomoea cavalcantei and I. marabaensis from Amazon Canga savannahs
Source: Sci Rep. 2017 Aug 8;7:7493. doi: 10.1038/s41598-017-07398-z (PMC5548896; doi:10.1038/s41598-017-07398-z)
Supplement: Supplementary file 1 — BABIYCHUK_SUPPLEMENTAL INFORMATION_SREP-16-51438B [file 41598_2017_7398_MOESM1_ESM.pdf]

Supplementary information

Natural history of the narrow endemics *Ipomoea cavalcantei* and *I. marabaensis* from Amazon Canga savannahs.

Elena Babiychuk[1,4*], Sergei Kushnir[1,4], Santelmo Vasconcelos[1], Mariana Costa Dias[1], Nelson Carvalho-Filho[1], Gisele Lopes Nunes[1], Jorge Filipe dos Santos[1], Lourival Tyski[2], Delmo Fonseca da Silva[2], Alexandre Castilho[3], Vera Lucia Imperatriz Fonseca[1] and Guilherme Oliveira[1]

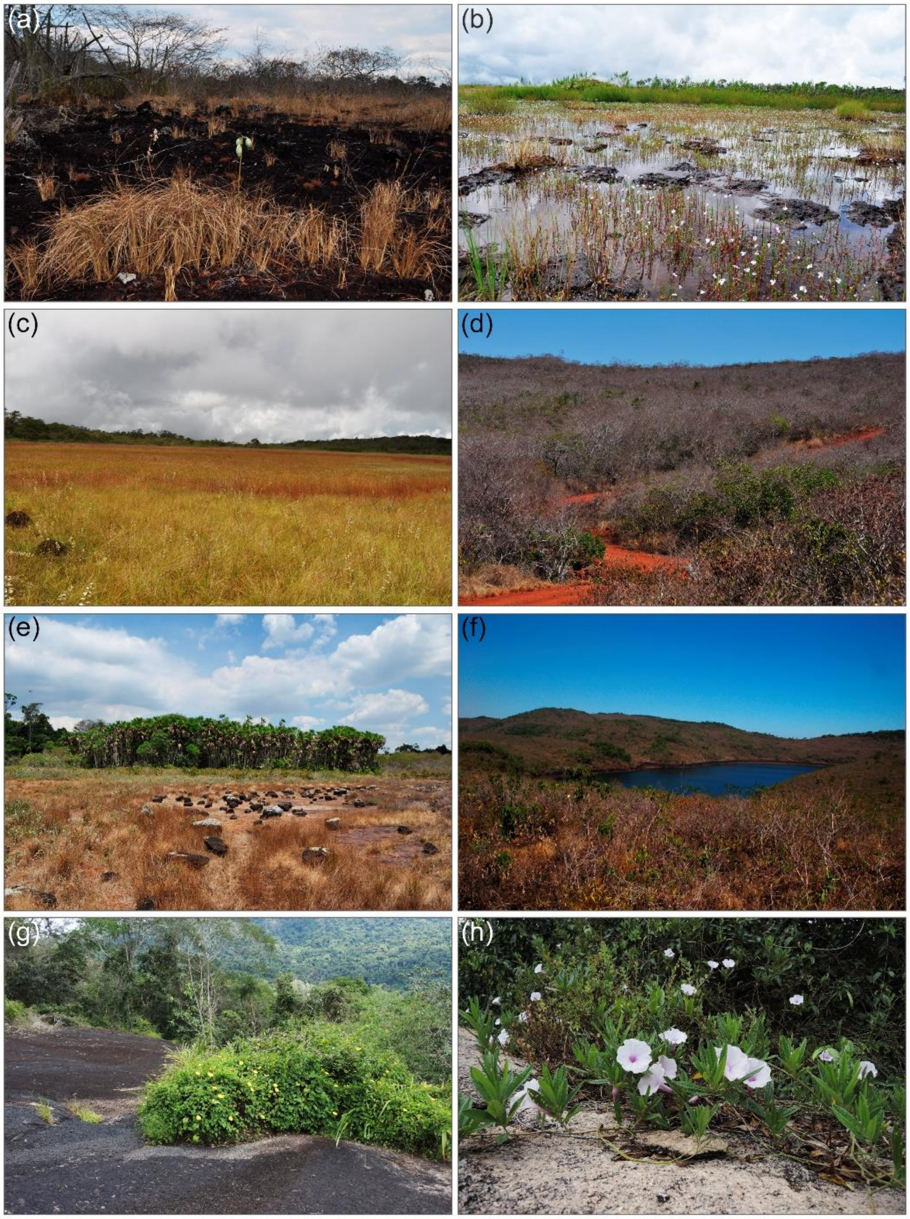

Supplementary Figure S1. Habitats of *I. cavalcantei* and *I. marabaensis*.

(a) Open barrens in Canga Tarzan at dry season. Fe-lateritic rocks are exposed and colored black most likely due to a cryptogamous cover, comprising cyanobacteria and lichens.
(b) Temporary water pools during wet season in Canga. Pools support herbaceous ephemeral plant communities.
(c) Canga grassland.
(d) Semi-deciduous shrubs and trees cover hilly S11 Plateau Canga. A band of the road is orange-red due to the intrinsic color of Fe-laterites.
(e) Semi-permanent shallow pond with Buritizal. A grove of buriti palms is where the water pool persists during dry season. Black boulders are Fe-lateritic rocks that are submerged in water during

wet season. Organic matter from dead vegetation residues of a wet season growth covers the landscape.

(f) A permanent lake Amendoim in S11 Plateau Canga during dry season. Shrubby vegetation cover the Fe-laterite hills.

(g) Inselberg Pedra do Gavião (Hawk`s Rock in Portuguese) in the Carajás National Forest. The grey is an exposed rock of the mountain. The mountain range on the background is covered by the mountain forest. A group of shrubs and climbers are clinging to the rock surface in a small depression. Convolvulaceae species *Operculina hamiltonii* used in this work is a part of this small plant community. Individuals of the species are in front, bright green bushes covered by bright yellow dots that are species flowers.

(h) Inselberg Sossego near the copper mining operation. This site is not within the Carajás National Forest. *I. marabaensis* is found among the rock-fringing vegetation. Thus, *I. marabaensis* can survive both in Canga and inselberg environments.

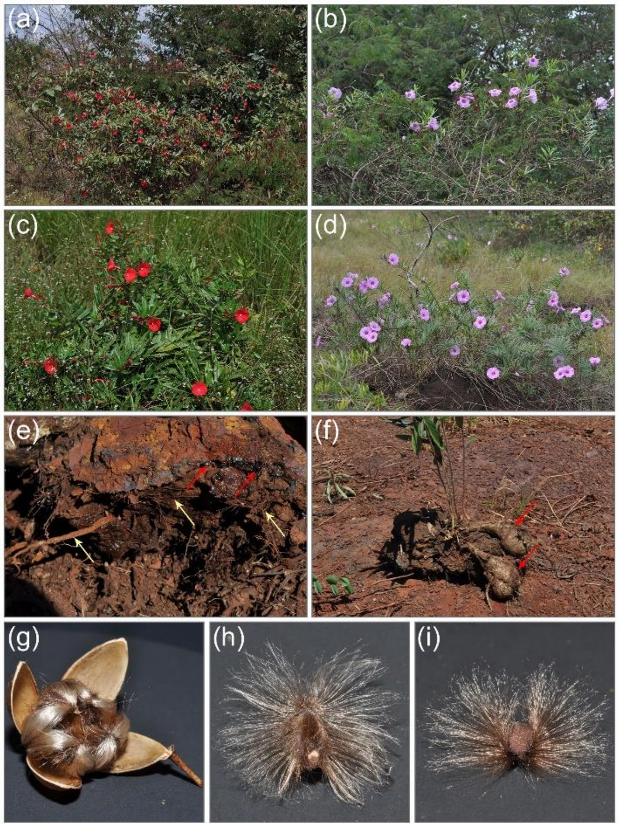

Supplementary Figure S2. *I. cavalcantei* and *I. marabaensis* share many morphological and ecophysiological traits.

(a), **(b), (c)**, **(d)**. Growth habits and flower color difference of Carajás morning glories. *I. cavalcantei* **(a,c)** and *I. marabaensis* **(b,d)** are both deciduous perennials, that could be found either as climbing lianas, when located in shrubby Canga habitats **(a,b)**, or grow as small shrubs with short shoot internodes, in open grassland or bare rock habitats **(c,d)**, indicating environmentally controlled developmental plasticity. The most distinct feature between the species are the flowers. *I. cavalcantei* flowers are usually colored deep red, have narrow tube and flattish limb of ca. 4-6 cm in diameter. *I. marabaensis* flowers are trumpet shaped with broad tube. Flower color and morphology variation is suggestive of pollinator-driven differentiation. We observed visitation of *I. cavalcantei* red flowers by hummingbirds and distinct species of bees. In contrast, several species of insects, such as beetles, flies, solitary bees and wasps were commonly found inside of *I. marabaensis* flowers, which correlated with the damage to flower tissues and organs, including stamens and pistils. Thus, florivory could affect the species reproductive success. Florivory of *I. cavalcantei* was less evident, indicating that florivory could have as well played a role in the evolvement of flower trait differentiation. The clonal propagation ability of the species is not known, but could be insignificant, because shoots do not trail over the land surface and have strong negative gravitropism. Flowering and seed set occurs during the rainy season between December and May.

(e), **(f)** Growth on Fe-laterite rocks. A single *I. cavalcantei* individual was excavated from Fe-lateritic rocks in a Canga N4 area. The bottom side of the excavated rock fragment **(e)** illustrates the roots of plants (yellow arrows). The black bands in the exposed rock surface are hematite, which is pure iron-oxides (red arrows). The root system of the species can penetrate the crevices and fissures in the lateritic rocks, and comprises tuberous roots. The same rock as in **(e)** with an embedded *I. cavalcantei* root system illustrates the tuberous roots of the species (red arrows). *I. marabaensis* develops similar storage organs. The root storage organs are likely to facilitate plant survival during the dry season and after fires. Species develop woody stems both as a secondary growth from the previous year's shoots, and from buds at the often exposed tuberous roots.

(g) Dry open fruit of *I. cavalcantei*. The fruit is a four locule dry capsule. Both species could produce four seeds per capsule.

(h), **(i)** Mature dry seeds from a fruit in **(g)**. Seeds are covered by well-developed trichomes (hairs). The same seed photographed from the ventral **(h)** and dorsal **(i)** sides. *I. marabaensis* seeds develops similar 5-7 mm long hairs. The trait is variable both in hair color, density and length and could facilitate seed dispersal by the wind and water torrents during rainy seasons, as originally proposed by Austin[11,12].

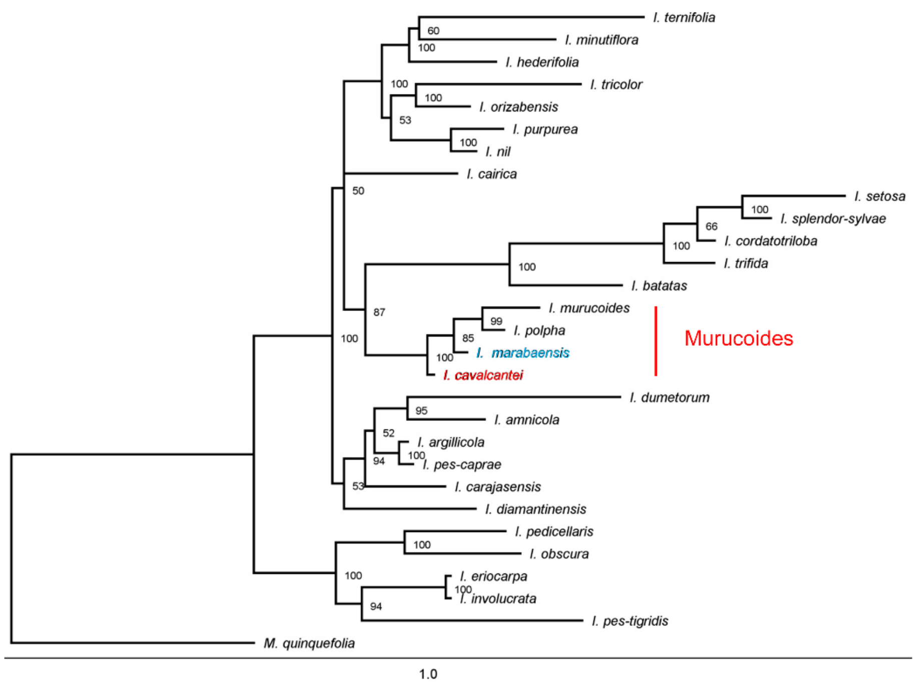

Supplementary Figure S3. Phylogenetic placement of *I. cavalcantei* and *I. marabaensis.*

The sequences of *rbcL*, *matK*, *rpoB*, *rpoC*, *psbK*, *atpF* and *psbA-trnH* from indicated species were aligned, trimmed and concatenated. The shown topology is from the analysis of the MUSCLE alignment in MrBayes. Numbers behind the nodes are Bayesian posterior probabilities. The sequences of *Merremia quinquefolia* were used to root the tree. The placement of *Ipomoea cavalcantei* and *I. marabaensis* within the *Murucoides* clade was supported by maximum parsimony analysis in PAUP (97%) and maximum likelihood analysis in PHYLM (99%) software packages.

(a)

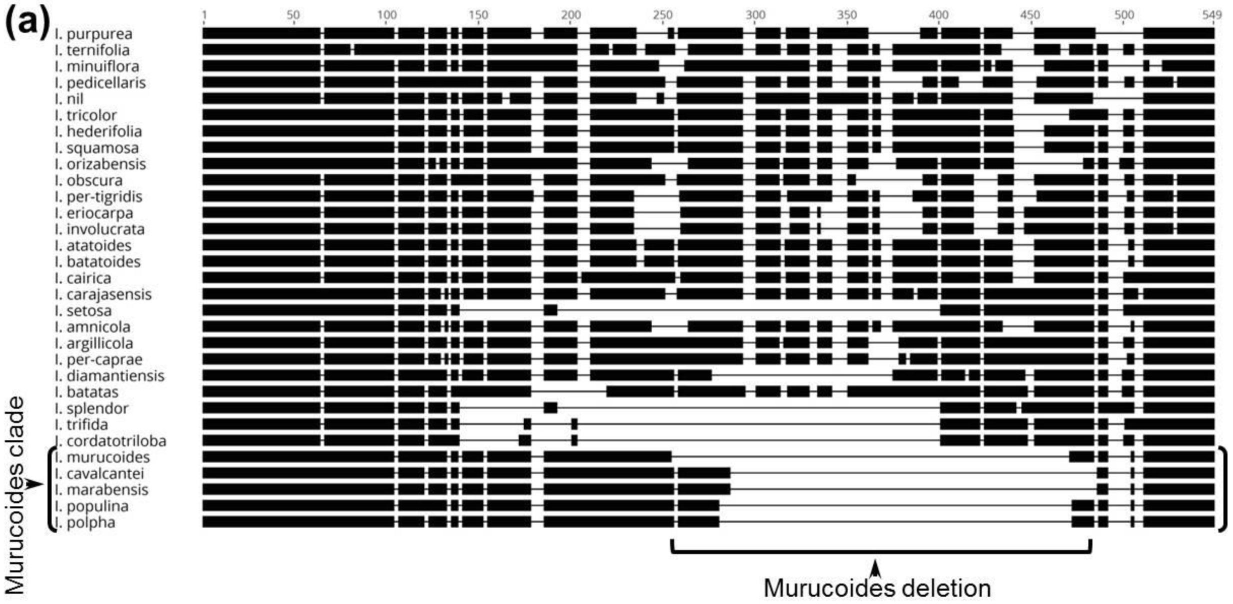

(b)

	I. murucoides	*I. cavalcantei*	*I. marabaensis*	*I. populina*	*I. polpha*
I. cordatotriloba	**54.155**	**47.146**	**47.146**	**50.503**	**50.63**
I. murucoides		**85.03**	**85.03**	**91.159**	**90.244**
I. cavalcantei			**100**	**93.393**	**93.976**
I. marabaensis				**93.393**	**93.976**
I. populina					**99.083**

Supplementary Figure S4. Distribution of deletion/insertions in the *psbA-trnH* intergenic spacers (IGS).

(a) Low resolution view of the alignment of available GenBank sequences. Alignment was generated with MUSCLE. Lines represent deletions/insertions. The Murucoides clade species *I. murucoides* and *I. polpha* are characterized by a large deletion. A very similar deletion of 158 bp as found in *I. cavalcantei/I. marabaensis*, as compared to *I. carajasiensis*, for example. This alignment indicates that *I. populina* could belong to the Murucoides clade.

(b) The sequence homology matrix of the alignment in **(a)**. Numbers are the percentage of sequence identity between a subset of species. This table illustrates high similarity between *I. murucoides, I. cavalcantei, I. marabaensis, I. polpha* and *I. populina* IGS sequences.

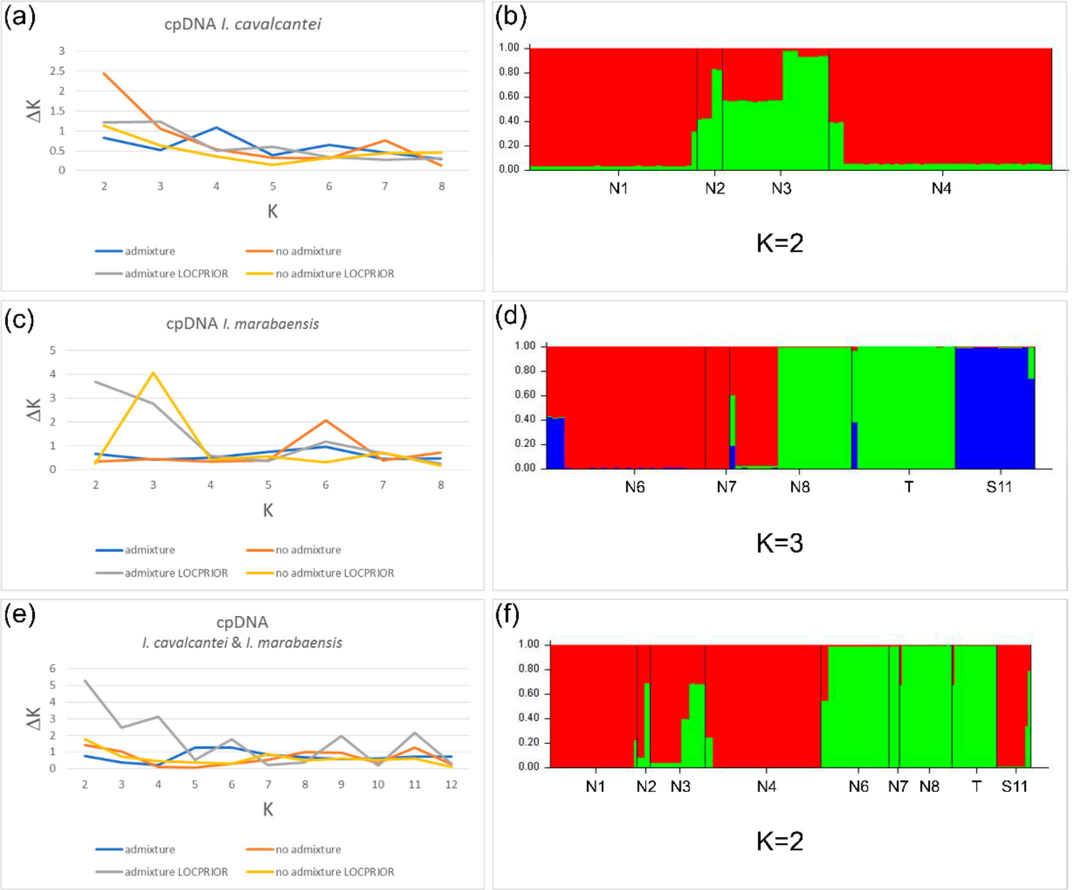

Supplementary Figure S5. STRUCTURE analysis of populations based on chloroplast DNA markers.

(a), (b) *I. cavalcantei***.** The chloroplast DNA (cpDNA) markers were considered as a single locus with 4 alleles, following the advice in a user manual of STRUCTURE v 2.3.4 for non-recombining regions when inheritance is uniparental and haploid, such as mitochondrial DNA (mtDNA) and Y chromosomes. The ΔK values were calculated based on results from four models: (i) admixture without consideration of the prior locations (admixture); (ii) no admixture without consideration of the prior locations (no admixture); (iii) admixture with consideration of the prior locations (admixture LOCPRIOR); (iv) no admixture with consideration of the prior locations (no admixture LOCPRIOR). Bar plot represents the membership coefficients (Q) of individual plants when K = 2. Individuals are sorted by the sampling locations that are indicated on the horizontal axis. Left to right: Cangas N1, N2, N3, N4.

(c), (d) *I. marabaensis*. Bar plot represents the membership coefficients (Q) of individual plants when K = 3. Sampling locations from left to right: Cangas N6, N7, N8, Tarzan (T) and S11 Plateau (S11).

(e), (f) Comparison of two morning glory species. K = 2.

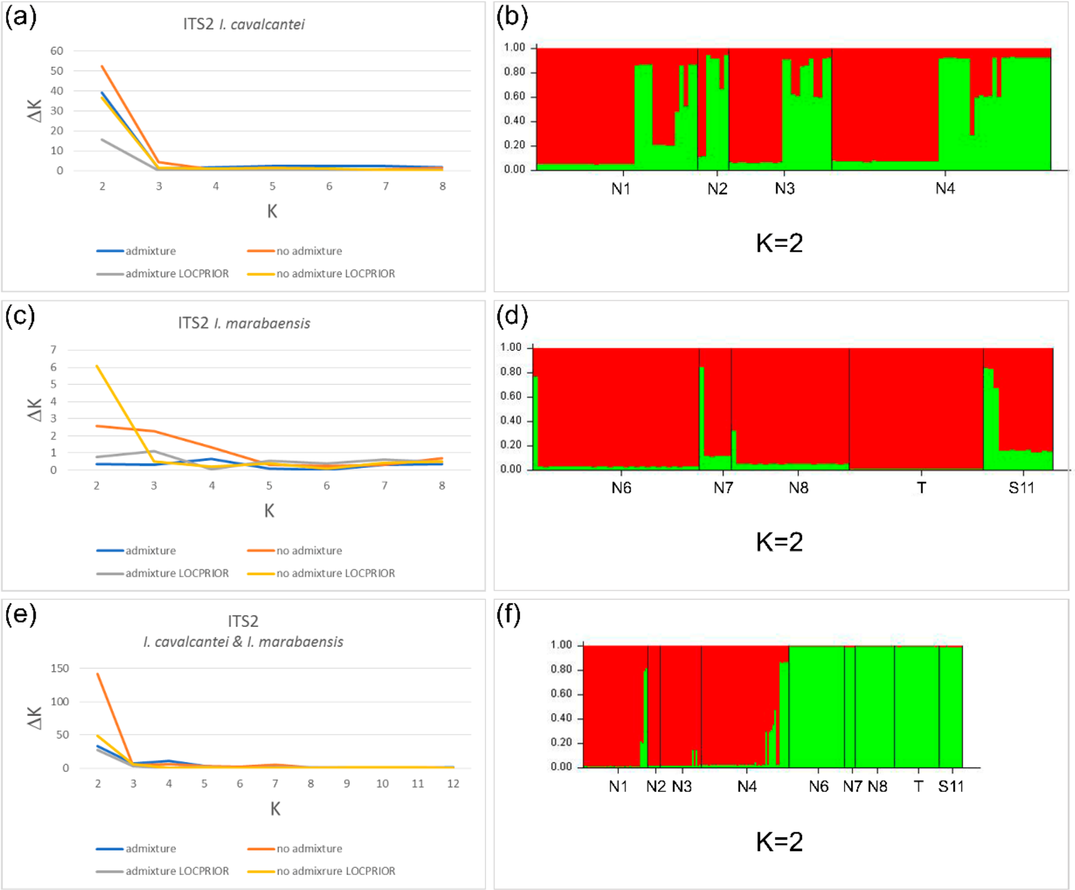

Supplementary Figure S6. STRUCTURE analysis of populations based on nuclear DNA ITS2 markers.

(a), (b) *I. cavalcantei* ITS2 single locus was represented by 12 alleles (Tables S4 and S5). The ΔK values were calculated based on results from four models: (i) admixture without consideration of the prior locations (admixture); (ii) no admixture without consideration of the prior locations (no admixture); (iii) admixture with consideration of the prior locations (admixture LOCPRIOR); (iv) no admixture with consideration of the prior locations (no admixture LOCPRIOR). Bar plot represents the membership coefficients (Q) of individual plants when K = 2. Individuals are sorted by the sampling locations that are indicated on the horizontal axis. Left to right: Cangas N1, N2, N3, N4. The cluster in red comprises all the individuals homozygous for the haplotype H2, whereas individuals homozygous or heterozygous for other 11 alleles were clustered separately (colored in green). Such clustering is unlikely to represent two different populations, maybe it could indicate autogamous mode of reproduction in the species.

(c), (d) *I. marabaensis* ITS2 single locus was represented by 3 alleles (Tables S4 and S5). Sampling locations from left to right: Cangas N6, N7, N8, Tarzan (T) and S11 Plateau (S11). K = 2.

(e), (f) *I. cavalcantei* and *I. marabaensis*. The ITS2 locus was represented by the 14 alleles. K = 2.

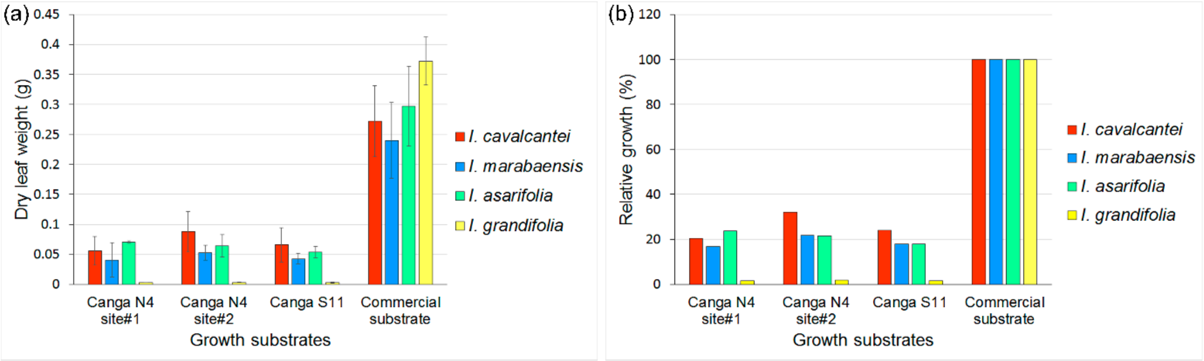

Supplementary Figure S7. Leaf biomass produced by the plants grown on different substrates.

(a) Leaf dry weight. Plants were grown as detailed in Methods. Leaves from ten-week-old plants were collected, dried for 72 hours at 65°C and the weight was measured (Dry leaf weight (g)). Four different substrates, soils, were tested. Soils samples from natural ecosystems were collected at two sites in Canga N4 (Canga N4 site#1; Canga N4 site#2); one site in Canga S11 Plateau (Canga S11). The growth substrate (commercial substrate) that is used for horticulture applications in Belém (Pará, Brazil) was purchased at a local horticulture supplier Yamanaka Comércio Ltda. The averages were plotted using measurements of nine replicates per soil tested. The exception was the Canga S11 Plateau soil tests in which six replicates per species were grown and analyzed. The data series are colored according to the analyzed species identities. *Merremia aegyptia* and *Operculina hamiltonii* were not analyzed, because the species seedlings were strongly inhibited in leaf production and all areal organs were necrotic. Error bars are standard deviations. The growth of the four analyzed species was inhibited on Canga soils, as compared to a commercial substrate.
(b) Relative growth inhibition on Canga soils. Dry leaf weight produced by plants grown on commercial substrate is expressed as 100%. This chart illustration suggests that in laboratory conditions *Ipomoea asarifolia* growth on Canga soils was comparable to the growth of Canga-adapted *I. cavalcantei* and *I. marabaensis*.

Supplementary Table S1. Sampling of the taxa present in surveyed Cangas.

Species	Canga	N	Longitude	Latitude	Altitude (m)
I. cavalcantei	N1	5	50° 17' 06"W	06° 01' 46"S	711
I. cavalcantei	N1	4	50° 17' 03"W	06° 02' 00"S	715
I. cavalcantei	N1	12	50° 17' 48"W	06° 00' 56"S	675
I. cavalcantei	N1	2	50° 16' 04"W	06° 02' 40"S	696
I. cavalcantei	N1	3	50° 16' 60"W	06° 01' 52"S	713
I. cavalcantei	N1	10	50° 17' 44"W	06° 00' 49"S	670
I. cavalcantei	N2	3	50° 15' 00"W	06° 03' 25"S	696
I. cavalcantei	N2	4	50° 15' 03"W	06° 03' 28"S	691
I. cavalcantei	N3	8	50° 12' 41"W	06° 02' 29"S	704
I. cavalcantei	N3	5	50° 12' 45"W	06° 02' 20"S	695
I. cavalcantei	N3	5	50° 12' 36"W	06° 02' 30"S	701
I. cavalcantei	N3	5	50° 12' 36"W	06° 02' 31"S	708
I. cavalcantei	N4	14	50° 11' 13"W	06° 06' 08"S	705
I. cavalcantei	N4	12	50° 11' 21"W	06° 05' 59"S	704
I. cavalcantei	N4	6	50° 11' 15"W	06° 06' 30"S	690
I. cavalcantei	N4	1	50° 11' 02"W	06° 06' 43"S	707
I. cavalcantei	N4	5	50° 11' 02"W	06° 06' 45"S	702
I. cavalcantei	N4	2	50° 11' 02"W	06° 06' 46"S	700
I. cavalcantei	N4	4	50° 11' 14"W	06° 06' 33"S	688
I. cavalcantei	N4	2	50° 11' 01"W	06° 06' 46"S	693
I. cavalcantei	N4	3	50° 11' 09"W	06° 06' 38"S	688
I. cavalcantei × marabaensis	N4	1	50° 11' 03"W	06° 06' 43"S	701
I. cavalcantei × marabaensis	N4	1	50° 11' 01"W	06° 06' 35"S	712
I. cavalcantei × marabaensis	N4	1	50° 04' 55"W	06° 03' 00"S	656
I. marabaensis	N4	1	50° 11' 07"W	06° 06' 10"S	720
I. marabaensis	N5	3	50° 08' 04"W	06° 07' 34"S	697
I. marabaensis	N6	4	50° 10' 32"W	06° 07' 45"S	699
I. marabaensis	N6	7	50° 10' 36"W	06° 07' 47"S	693
I. marabaensis	N6	8	50° 10' 37"W	06° 07' 50"S	695
I. marabaensis	N6	5	50° 10' 33"W	06° 07' 50"S	696
I. marabaensis	N6	7	50° 10' 34"W	06° 07' 48"S	697
I. marabaensis	N7	1	50° 10' 27"W	06° 09' 12"S	683
I. marabaensis	N7	3	50° 10' 29"W	06° 09' 10"S	685
I. marabaensis	N7	2	50° 10' 33"W	06° 09' 08"S	679
I. marabaensis	N8	7	50° 09' 16"W	06° 10' 15"S	721
I. marabaensis	N8	6	50° 09' 07"W	06° 10' 22"S	711
I. marabaensis	N8	5	50° 09' 46"W	06° 09' 54"S	696
I. marabaensis	N8	2	50° 08' 49"W	06° 10' 45"S	681
I. marabaensis	N8	2	50° 08' 07"W	06° 11' 07"S	675
I. marabaensis	S11	2	50° 26' 55"W	06° 20' 58"S	722
I. marabaensis	S11	2	50° 23' 06"W	06° 22' 57"S	742
I. marabaensis	S11	4	50° 22' 24"W	06° 23' 43"S	727
I. marabaensis	S11	5	50° 22' 22"W	06° 23' 49"S	718
I. marabaensis	T	7	50° 09' 54"W	06° 19' 57"S	720
I. marabaensis	T	9	50° 09' 53"W	06° 20' 12"S	813
I. marabaensis	T	5	50° 08' 51"W	06° 20' 05"S	726
I. marabaensis	T	4	50° 08' 36"W	06° 19' 54"S	695
I. marabaensis	SO	1	50° 05' 20"W	06° 27' 47"S	269

Abbreviations. N - number of individuals analyzed. The habitat of population Sossego (SO) is not in a Canga, but on a granitic inselberg.

Supplementary Table S2. Natural variation in *I. cavalcantei* and *I. marabaensis* plastomes.

**Gene**	***psbK-psbI***	***rpoB***	***rbcL***	***matK***	***atpF-atpH***	***rpoC1***[a]	***psbA-trnH***[a]
Aligned sequences (bp)	315	481	568	620	446	450	288
Number of individuals:							
I. cavalcantei	7	17	117	19	5	103	103
I. marabaensis	3	5	86	4	4	83	83
Sequence identity[a]	100%	100%	100%	100%	100%	99-100%	98-100%

[a] The *rpoC1* gene and *psbA-trnH* intergenic region showed intra(inter)specific sequence variation, which was assembled as plastid haplotypes (See Supplementary Table S3). Other sequences were identical between and within the species, i.e. 100%.

Supplementary Table S3. Plastome types.

**SNP**[b]			***I. cavalcantei***[c]				***I.marabaensis***[c]							**T**[d]
**Plastome**[a]	***rpoC1***	***psbA-trnH***	**N1**	**N2**	**N3**	**N4**	**N5**	**N6**	**N7**	**N8**	**T**	**S11**	**SO**	
AT	A	TTT	32	3	12	41		3		1	1	11		104
AA	A	AAA			3							1		4
CT	C	TTT	1	2	6	3	2	23	4	7				48
CA	C	AAA								12	16	1	1	30

[a] Identification of plastomes based on *rpoC1* and *psbA-trnH* polymorphic sites.
[b] Bases at polymorphic sites (SNP). The SNP in *rpoC1* is non-synonymous and result in an inferred difference in the amino acid residue sequences of the rpoC1 protein, i.e. glutamine (Q) *versus* lysine (K). The polymorphism in *psbA-trnH* amplicon is in an intergenic transcribed region.
[c] Analyzed species; the areal distributions, i.e. Canga identifiers (Fig. 1b), and the number of individuals of a particular plastome type are shown.
[d] Total number of plastome types/haplotypes found at all locations.

Supplementary Table S4. The estimates of plastome haplotype and nucleotide diversities, neutrality tests results and haplotype frequencies analysis.

**Populations**	**N**	**H**	**S**	***h***	***π***	**Neutrality test**		**Chakraborty`s test**	
						**Tajima`s**	**Fu`s**	**No of haplotypes**	
						D	F_S	**Exp.**	**Obs.**
I. cavalcantei, N1	33	2	1	0.06	0.00007	-1.14	-1.29	1.2	2
I. cavalcantei, N2	5	2	1	0.60	0.00068	1.22	0.63	2.4	2
I. cavalcantei, N3	21	3	4	0.60	0.00136	0.22	1.73	3.9	3
I. cavalcantei, N4	44	2	1	0.13	0.00015	-0.60	-0.30	1.5	2
I. marabaensis, N6	26	2	1	0.21	0.00024	-0.30	0.16	1.7	2
I. marabaensis, N7	4	1	0	0	0	NA	NA	NA	NA
I. marabaensis, N8	20	3	4	0.54	0.00183	1.25	2.51	3.4	3
I. marabaensis, T	17	2	4	0.12	0.00053	-1.84*	1.18	1.3	2
I. marabaensis, S11	13	3	4	0.29	0.00113	-0.76	0.77	1.8	3

Abbreviations. N - sample size, H - number of alleles/haplotypes, S - number of polymorphic sites, *h* – haplotype diversity, π – nucleotide diversity. Significance levels: *p < 0.05.

Supplementary Table S5. Genotypes of plants identified by the sequences of ITS2 amplicons.

			I. cavalcantei[d]				*I.marabaensis*[d]								T[e]
**Type**[a]	**SNP**[b]	**HP**[c]	**N1**	**N2**	**N3**	**N4**	**N4**	**N5**	**N6**	**N7**	**N8**	**T**	**S11**	**SO**	
L	G-C-T-G-G-C	H2	22	2	12	24									60
K	G-C-T-G-G-A	H3	2		2	5									9
J	G-C-T-C-G-C	H4	2	1		1									4
I	A-C-T-G-G-C	H5			2										2
H	A-C-T-C-G-C	H6				1									1
Y	R-C-T-G-G-C	H2 + H5	5			1									6
D	G-C-T-G-G-M	H2 + H3	1			4									5
V	R-C-T-G-G-M	H5 + H3		2	2										4
T	G-Y-T-S-G-M	H3 + H1	1		1	1									3
Z	G-C-T-S-G-C	H2 + H4		1	1	1									3
Q	G-Y-T-C-G-C	H4 + H1				2									2
S	G-Y-T-S-G-C	H2 + H1	1		1										2
O	G-T-T-S-G-C	H1 + H7				1									1
P	G-T-T-S-G-M	H1 + H9				1									1
R	G-Y-T-G-G-M	H3 + H7		1											1
N	G-C-T-S-G-M	H3 + H4			1										1
M	G-C-T-K-G-M	H3 + H8				1									1
W	R-Y-T-G-G-M	H3 + H10				1									1
X	C-C-T-S-G-C	H11 + H12			1										1
A	G-T-T-C-G-C	H1	2			5	1	3	30	5	21	25	10	1	103
C	G-T-C-C-G-C	H13								1			1		2
E	G-T-T-C-T-C	H14							1				1		2
F	G-T-Y-C-G-C	H1 + H13									1		1		2

[a] Arbitrary identification of types of individuals based on sequences of ITS2 amplicons.
[b] Polymorphic sites (SNP) at positions 156, 160, 165, 191, 207 and 215 numbered from 5` end of ITS2 in sequences (see Fig. 4). Gaps (-) are stretches of identical not shown sequences. The IUPAC nomenclature for the ambiguous reads: R = A/G; M = C/A; S = G/C; Y = C/T.
[c] Haplotype phasing (HP). Plant types without ambiguities are likely to have a homogeneous gametic state, i.e. are homozygous for a particular haplotype. Sequences with ambiguities could be a result of hybridization. Column shows our interpretation of hybridization to resolve ambiguities. Letters and numbers correspond to proposed haplotype composition of *I. cavalcantei* and *I. marabaensis* (Supplementary Table S6).
[d] Analyzed species; the areal distributions, i.e. Canga identifiers; and the number of individuals of a particular type are shown.
[e] Total numbers of plant types at all locations.

Supplementary Table S6. ITS2 Haplotypes.

		I. cavalcantei[b]				***I. marabaensis***[b]								N[c]
**Haplotype**	**SNP**[a]	**N1**	**N2**	**N3**	**N4**	**N4**	**N5**	**N6**	**N7**	**N8**	**T**	**S11**	**SO**	
H1	G-T-T-C-G-C	6		2	15	2	6	60	10	43	50	21	2	217
H2	G-C-T-G-G-C	51	5	26	54									136
H3	G-C-T-G-G-A	6	3	8	17									34
H4	G-C-T-C-G-C	4	3	2	5									14
H5	A-C-T-G-G-C	5	2	6	1									14
H6	A-C-T-C-G-C				2									2
H7	G-T-T-G-G-C		1		1									2
H8	G-C-T-T-G-C				1									1
H9	G-T-T-G-G-A				1									1
H10	A-T-T-G-G-C				1									1
H11	C-C-T-G-G-C			1										1
H12	C-C-T-C-G-C			1										1
H13	G-T-C-C-G-C								2	1		3		6
H14	G-T-T-C-T-C							2				2		4

[a] Polymorphic sites (SNP) at positions 156, 160, 165, 191, 207 and 215 numbered from 5` end of ITS2 sequences. Gaps (-) are stretches of identical not shown sequences. See Fig. 6 for more details.

[b] Observed frequencies of the haplotypes/chromosomes across Canga islands.

[c] Total observed frequencies of haplotypes.

Supplementary Table S7. The estimates of ITS2 haplotype and nucleotide diversities and neutrality test results.

Populations	N	H	S	He obs	He exp	*h*	π	Neutrality tests	
								Tajima`s *D*	Fu`s F_S
I. cavalcantei, N1	36	5	4	0.22	0.48	0.483	0.0031	-0.3617	-0.8451
I. cavalcantei, N2	7	5	4	0.57	0.81	0.813	0.0051	-0.3298	-1.3219
I. cavalcantei, N3	23	7	4	0.3	0.64	0.643	0.0039	-0.0711	-2.5399
I. cavalcantei, N4	49	10	4	0.26	0.65	0.646	0.0047	0.7105	-3.7627^{*}
I. marabaensis, N6	31	2	1	0	0.06	0.064	0.0003	-0.8939	-1.0703
I. marabaensis, N7	6	2	1	0	0.30	0.303	0.0014	-0.1949	0.2973
I. marabaensis, N8	22	2	1	0.05	0.04	0.045	0.0002	-1.1153	-1.5295
I. marabaensis, T	25	1	0	0	0	0	0	NA	NA
I. marabaensis, S11	13	3	2	0.08	0.34	0.341	0.0016	-0.6706	-0.6491

Abbreviations. N - sample size, H - number of alleles/haplotypes, S - number of polymorphic sites, He obs - observed heterozygosity, He exp - expected heterozygosity, *h* – haplotype diversity, π – nucleotide diversity. Significance levels: $^{*}p < 0.05$.

Supplementary Table S8. Nuclear genome size (pg of DNA) determined by flow cytometry.

	N	Mean ± s.d.	Min.	Max.
I. cavalcantei	31	2.46±0.1	2.22	2.61
I. marabaensis	43	2.47±0.05	2.34	2.58
I. cavalcantei × *marabaensis*	3	2.34±0.05	2.28	2.45

Abbreviations. N – number of analyzed individuals. The range of the measured values from the minimum (Min.) to maximum (Max.).

Supplementary Table S9. Nutrient content and physicochemical characteristics of soils[a].

Soil variables	Soil samples			
	**Canga N4 site#1**	**Canga N4 site#2**	**Canga S11 Plateau**	**Control**
pH in water	4.8±0.16	4.26±0.3	5.6±0.25	5.3±0.1
pH in $CaCl_2$	3.8±0.12	3.5±0.14	4.6±0.2	4.9±0.1
Organic matter content, O.M. (dag/Kg)	4.7±0.25	4.8±0.16	4.6±0.1	4.9±0.1
Organic C (%)	2.7±0.12	2.8±0.08	2.7±0.05	2.8±0.05
P extractable by anion exchange resin (mg/dm³)	42.2±0.25	14.1±2.1	6.1±1.1	177.5±15.6
P by Mehlich 1 extraction (mg/dm³)	21.2±0.6	5±0.7	3.16±0.2	170.7±16.4
K (mg/dm³)	42±3	46±2	137±3	356±39
S (mg/dm³)	6.3±0.2	6.4	9.7±0.6	127.5±36.6
Ca^{+2} ($cmol_c/dm^3$)	0.1	0.9±0.21	1.4±0.12	6.9±0.4
Mg^{+2} ($cmol_c/dm^3$)	0.1	0.3±0.08	0.7±0.05	3.9±0.25
Al^{+3} ($cmol_c/dm^3$)	2.43±0.05	0.93±0.26	0.23±0.09	0.1
Na^{+} ($cmol_c/dm^3$)	<0.1	<0.1	0.1	0.13±0.05
H+A I, soil potential acidity ($cmol_c/dm^3$)	21.3±1.1	23±3.1	9.1±0.6	6.2±0.2
CEC, cation exchange capacity ($cmol_c/dm^3$)	21.7±1.1	24.5±3.19	11.7±0.45	18.2±0.8
BS, base saturation (%)	2	5.6±0.94	21.6±2	65.3±1.7
M, aluminum saturation (%)	85.6±0.5	40±7.8	8.3±3	1±0
**Ratios of Ca, Mg and K contents**				
Ca/Mg	1	3.1	2.17	1.76
Ca/K	0.93	7.6	4.1	7.7
Mg/K	0.93	2.5	1.93	4.3
**Saturation of the Exchange Complexes**				
K (%)	0.33±0.5	1	3	5
Ca (%)	0	3.6±0.9	12.3±1.2	38.3±1.3
Mg (%)	0	1.3±0.5	5.7±0.5	21.7±0.5
Na (%)	0	0	1	1
H+Al (%)	99.6±0.5	94.7±1.2	78±1.6	34±1.6
**Micronutrients**				
B (mg/dm³)	0.2	0.7±0.3	0.23±0.1	0.86±0.05
Zn (mg/dm³)	1.7±0.1	3.6±0.3	2.9±0.1	11.6±2.5
Fe (mg/dm³)	553±6	496±38	581±4	550±46
Mn (mg/dm³)	4.5±0.1	29±1.2	22.5±1.4	54±10
Cu (mg/dm³)	0.16±0.05	0.3	0.56±0.05	1.3±0.1
**Granulometric analysis**				
Silt (%)	37	24	23	33
Clay (%)	33	16	21	12
Sand (%)	30	60	56	55

[a] The measurements were done in triplicate. Variation (±) from the average is indicated in standard deviations.

Supplementary Table S10. Identification of Convolvulaceae species used in the common garden soil transplantation experiments.

	ITS2[b]	***psbA-trnH***[b]	***rpoC***[b]	***rpoB***[b]	***rbcL***[b]	***matK***[b]	***psbK***[b]	***atpF***[b]
**Tentative identification /likely identity**[a]								
Ipomoea asarifolia[c,d]/<br>*I. asarifolia* (*ITS2*)	465<br>314/314=100%<br>Gaps=0<br>*I. asarifolia*	321<br>316/342(92%)<br>Gaps=21<br>*I. batatas*	507<br>505/505=100%<br>Gaps=0<br>*I. nil*<br>*I. batatas*<br>*I. minutiflora*<br>*I. cordatotriloba*<br>*I. trifida*<br>*I. splendor-sylvae*<br>*I. setosa*<br>*I. polpha*<br>*I. pes-caprae*<br>*I. pedicellaris*	NA	735<br>735/735=100%<br>Gaps=0<br>*I. leptophylla*<br>*I. involucrata*<br>*I. eriocarpa*<br>*Turbina oblongata*	NA	NA	NA
Merremia aegyptia[d]/<br>*Merremia* spp. (*ITS2, psbA-trnH, rpoC, rpoB, rbcL, matK*)	446<br>411/456=90%<br>Gaps=12<br>*I. macrantha*	439<br>418/419=99%<br>Gaps=0<br>*M. aegyptia*	502<br>501/501=100%<br>Gaps=0<br>*M. quinquefolia*	484<br>478/480(99%)<br>Gaps=0<br>*M. quinquefolia*	747<br>745/747=99%<br>Gaps=0<br>*M. quinquefolia*	777<br>775/777=99%<br>Gaps=0<br>*M. quinquefolia*	NA	NA
Operculina hamiltonii[e]/<br>*Operculina* spp. (*psbA-trnH, rpoC, rpoB, rbcL, matK, psbK, atpF*)	470<br>419/470(89%)<br>Gaps=13<br>*I. macrantha*	546<br>525/546(96%)<br>Gaps=17<br>*O. macrocarpa*	496<br>494/494=100%<br>Gaps=0<br>*O. macrocarpa*	484<br>480/480(100%)<br>Gaps=0<br>*O. macrocarpa*	737<br>736/737=99%<br>Gaps=0<br>*O. macrocarpa*	783<br>780/783(99%)<br>Gaps=1<br>*O. macrocarpa*	379<br>379/379=100%<br>Gaps=0<br>*O. macrocarpa*	487<br>486/487=99%<br>Gaps=0<br>*O. macrocarpa*
Ipomoea grandifolia.[f]/<br>*Ipomoea* spp. (*ITS2, rpoC, rpoB, rbcL, matK, psbK, atpF*)	453<br>390/393=99%<br>Gaps=1<br>*I.batatas*	NA	485<br>483/483=100%<br>Gaps=0<br>*I. batatas*<br>*I. trifida*<br>*I. nil*<br>*I. cordatotriloba*<br>*I. minutiflora*<br>*I. splendor-sylvae*	495<br>489/491=99%<br>Gaps=0<br>*I. cordatotriloba*	749<br>749/749=100%<br>Gaps=0<br>*I. batatas*<br>*I. trifida*<br>*I. cordatotriloba*	741<br>741/741=100%<br>Gaps=0<br>*I. batatas*<br>*I. trifida*<br>*I. cordatotriloba*	397<br>397/398=99%<br>Gaps=1<br>*I. cordatotriloba*	486<br>486/486=100 %<br>Gaps=0<br>*I. batatas*<br>*I. trifida*

[a] Species were identified in the field and seeds were collected. To validate species identification further, tissue samples from seedling grown in the laboratory were used to extract DNA; generate DNA amplicons and obtain amplicon sequences. Based on results of the blast-n search, we propose molecular species identification after the slash (/). In brackets, are the gene/loci names based on which we propose species/genus level molecular identification.

[b] NA – no sequences available from the query species in this study. *ITS2* is a transcribed rDNA spacer; other genes are from the plastomes. In the Table cells for the gene columns, the first number is the length of the query sequence analyzed by blast-n at NCBI. Follows, the range of matching sequences and the percentage of similarity/identify. Gaps – number of gaps in the alignments. For alignments that showed 100% identity over the widest range, the all species sources are listed by botanical names. For alignment with <100% identity only the top hit is indicated.

[c] *Ipomoea asarifolia* sequences in GenBank are available only for two loci. *ITS2* and granule-bound starch synthase.

[d] *I. asarifolia* and *Merremia aegyptia* originate from rocky environments at Brazilian Atlantic coast. *M. aegyptia* is a climber, often forming dense cover over shrubs. *I. asarifolia* was trailing liana that often grew on exposed rock surfaces. Both species could be aggressive weeds, and are common at disturbed sites.

[e] *Operculina hamiltonii*[14] was collected on a granitic inselberg at Carajás National Forest. Individuals grew in a small depression in an exposed granitic mountain side, indicating tolerance to high heat and sun light intensity; an ability to survive with little nutrients, which is similar to most of Canga environments. We have never observed *Operculina hamiltonii* in undisturbed Canga habitats.

[f] *Ipomoea grandifolia* is a species that is common in wet biomes of the Carajás National Forest. Species are aggressive climbers. In several areas, especially along the roads, plants would completely cover the shrubs and trees up to a height 5-6 meters. We have never observed *I. grandifolia* in Canga undisturbed habitats.

Supplementary Table S11. Primer sequences used to amplify and sequence the analyzed partial gene regions and intergenic spacers in *I. cavalcantei* and *I. marabaensis*.

Gene loci	Primer name	Primer sequence (5’-3’)	Tm, $^oC^e$
rpoB[a]	rpoB-1f	AAGTGCATTGTTGGAACTGG	53.7
	rpoB-4r	GATCCCAGCATCACAATTCC	53.7
rpoC1[a]	rpoC1-1f	GGCAAAGAGGGAAGATTTCG	53.8
	rpoC1-4r	CCATAAGCATATCTTGAGTTGG	51.1
rbcL[b]	rbcL-Sf	AGACCTTTTTGAAGAAGGTTCTGT	54.6
	rbcL-Sr	TCGGTCAGAGCAGGCATATGCCA	62.6
matK[c]	3F_KIM	CGTACAGTACTTTTGTGTTTACGAG	53.9
	1R_KIM	ACCCAGTCCATCTGGAAATCTTGGTTC	60.4
atpF-atpH[c]	atpF	ACTCGCACACACTCCCTTTCC	59.6
	atpH	GCTTTTATGGAAGCTTTAACAAT	50.1
psbK-psbI[c]	psbK	TTAGCCTTTGTTTGGCAAG	51.1
	psbI	AGAGTTTGAGAGTAAGCAT	48
psbA-trnH[c]	psbA	GTTATGCATGAACGTAATGCTC	52.3
	trnH	CGCGCATGGTGGATTCACAATCC	61.1
ITS2[d]	S2F	ATGCGATACTTGGTGTGAAT	51.9
	S3R	GACGCTTCTCCAGACTACAAT	54.2

[a] Primers recommended by the Royal Botanic Gardens, Kew, UK (2007-onwards) DNA Barcoding, Phase 2 Update. Web page: http://www.kew.org/barcoding/update.html.
[b] Primers from Dong, W. *et al.* Discriminating plants using the DNA barcode rbcLb: an appraisal based on a large data set. *Mol. Ecol. Res.* **14**, 336-343 (2014).
[c] Unpublished primers from Ki-Joong Kim, School of Life Sciences and Biotechnology, Korea University, Seoul, Korea. kimkjkorea.ac.kr.
[d] Primers from Chen, S. *et al.* Validation of the ITS2 region as a novel DNA barcode for identifying medicinal plant species. *PLoS ONE* **5**, e8613 (2010).
[e] Melting temperatures (Tm) were calculated with OligoAnalyzer Version 3.1 (https://www.idtdna.com/calc/analyzer). In PCR reactions, the primer annealing temperature was 54 °C (except for the *matK* amplicons for which the annealing temperature was 46 °C) as detailed in Methods.
